# Genomic analyses suggest adaptive differentiation of northern European native cattle breeds

**DOI:** 10.1111/eva.12783

**Published:** 2019-03-12

**Authors:** Astrid V. Stronen, Cino Pertoldi, Laura Iacolina, Haja N. Kadarmideen, Torsten N. Kristensen

**Affiliations:** ^1^ Section of Biology and Environmental Science, Department of Chemistry and Bioscience Aalborg University Aalborg Denmark; ^2^ Department of Biology, Biotechnical Faculty University of Ljubljana Ljubljana Slovenia; ^3^ Department of Biotechnology and Life Sciences Insubria University Varese Italy; ^4^ Aalborg Zoo Aalborg Denmark; ^5^ Quantitative Genomics, Bioinformatics and Computational Biology Group, Department of Applied Mathematics and Computer Science Technical University of Denmark Kongens Lyngby Denmark

**Keywords:** animal health, artificial selection, *Bos taurus*, climate adaptation, conservation genomics, environmental selection, production traits, single nucleotide polymorphism

## Abstract

Native domestic breeds represent important cultural heritage and genetic diversity relevant for production traits, environmental adaptation and food security. However, risks associated with low effective population size, such as inbreeding and genetic drift, have elevated concerns over whether unique within‐breed lineages should be kept separate or managed as one population. As a conservation genomic case study of the genetic diversity represented by native breeds, we examined native and commercial cattle (*Bos taurus*) breeds including the threatened Danish Jutland cattle. We examined population structure and genetic diversity within breeds and lineages genotyped across 770K single nucleotide polymorphism loci to determine (a) the amount and distribution of genetic diversity in native breeds, and (b) the role of genetic drift versus selection. We further investigated the presence of outlier loci to detect (c) signatures of environmental selection in native versus commercial breeds, and (d) native breed adaptation to various landscapes. Moreover, we included older cryopreserved samples to determine (e) whether cryopreservation allows (re)introduction of original genetic diversity. We investigated a final set of 195 individuals and 677K autosomal loci for genetic diversity within and among breeds, examined population structure with principal component analyses and a maximum‐likelihood approach and searched for outlier loci suggesting artificial or natural selection. Our findings demonstrate the potential of genomics for identifying the uniqueness of native domestic breeds, and for maintaining their genetic diversity and long‐term evolutionary potential through conservation plans balancing inbreeding with carefully designed outcrossing. One promising opportunity is the use of cryopreserved samples, which can provide important genetic diversity for populations with few individuals, while helping to preserve their traditional genetic characteristics. Outlier tests for native versus commercial breeds identified genes associated with climate adaptation, immunity and metabolism, and native breeds may carry genetic variation important for animal health and robustness in a changing climate.

## 1 INTRODUCTION

Domestication of plants and animal species has permitted significant human population growth and the development of modern human societies (Larson & Burger, 2013; Larson & Fuller, 2014; Marshall, Dobney, Denham, & Capriles, 2014). Domestication is considered to have occurred along three major pathways, termed commensal, prey or directed (reviewed in Larson & Burger, 2013; Larson & Fuller, 2014). They describe the commensal pathway as centred on animal habituation to a human niche, whereas the prey pathway involved animals that humans initially preyed upon and later started to manage. In contrast, Larson and Burger (2013) and Larson and Fuller (2014) note that the more recent directed pathway has been the only deliberate route to domestication, which bypassed the habituation and management phases. There is evidence that sheep (*Ovis *sp.), goats (*Capra *sp.) and cattle (*Bos *sp.) were domesticated 10,500–10,000 years before present via the prey pathway, through various stages of intensive breeding of captive animals and the subsequent development of distinct breeds (Larson & Burger, 2013; Larson & Fuller, 2014). Conservation of native domestic animals and plants is now receiving growing attention. Populations of conservation concern may encompass important cultural heritage and potentially genetic diversity relevant for modern breeding and future food security such as for production traits, adaptation to harsh environments and climate change (Hoffmann, 2013; Iacolina et al., 2016; Kantanen et al., 2015; Kristensen, Hoffmann, Pertoldi, & Stronen, 2015).

### 1.1 Genetic diversity within and among native breeds

Native domestic breeds are known to share several conservation concerns with populations of wild species at risk, such as low effective population size (*N*_E_), which in turn reduces the effectiveness of selection and increases the impacts of genetic drift and inbreeding (Kantanen et al., 2000; Leroy et al., 2013; Pertoldi et al., 2014; Taberlet et al., 2008). Numerous livestock breeds have gone extinct or are threatened (FAO, 2007, 2015). Genetic variation within and between breeds is rapidly lost and, for many breeds, we have little information about levels of genetic variation, *N*_E_, and adaptation to past and present local environmental conditions. Past selection for conditions such as the ability to survive on food with limited nutritional content could be important for adaptation to climate change, and for the use of native breeds in habitat management. To maintain sustainable populations in the short term, an *N*_E_ of at least 50 is sometimes recommended in conservation genetics to keep inbreeding rates at acceptable levels, and an *N*_E_ >500 is needed to allow maintenance of evolutionary potential over time (i.e., across hundreds of generations) (Frankham, Bradshaw, & Brook, 2014; Hoffmann, Sgrò, & Kristensen, 2017). Because domestic animal breeds typically have *N*_E_ <100 (Leroy et al., 2013), current breeding practices raise long‐term concerns for the evolutionary potential of many of these populations.

Genomic methods and reproductive techniques are rapidly developing and can help provide answers to many important questions in conservation genetics. These include levels of inbreeding and genetic drift, genetic uniqueness, identification of genomic regions under selection, and creation of genetic rescue programmes or breeding schemes to maintain adaptive genetic variation in domestic animals more efficiently than pedigree‐based breeding (Kantanen et al., 2015; Kukučková et al., 2017; Porto‐Neto et al., 2014; Williams et al., 2015). Improved communication between the fields of research and management concerning experimental results on genetic rescue and other conservation actions is therefore important for an efficient management of populations with small and declining *N*_E_ (Hoffmann, Merilä, & Kristensen, 2016; Kristensen et al., 2015).

**Figure 1:**
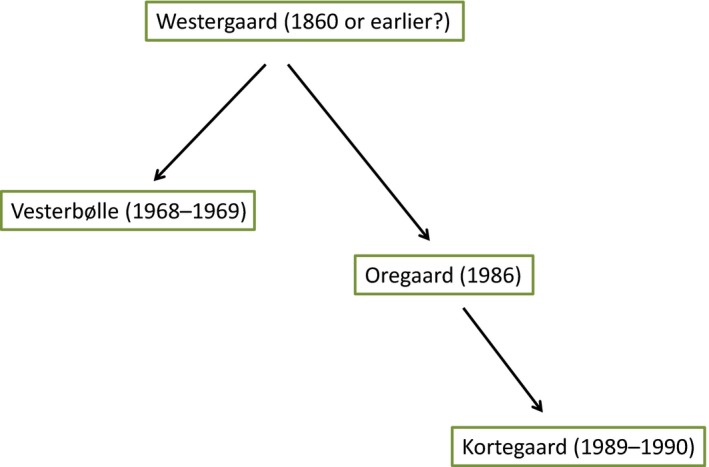
Proposed timeline for founding of the four contemporary lineages of the Danish Jutland cattle breed. The Westergaard‐lineage is deemed to be the oldest, although the precise time of its origin is unknown

An example of a native cattle (*Bos taurus*) breed under threat is the Danish Jutland cattle, which includes four contemporary lineages (within‐breed subpopulations maintained in (relative) isolation from other such groups). These are the Westergaard‐, Vesterbølle‐, Oregaard‐ and Kortegaard‐lineages (Figure 1). An earlier microsatellite genetic study of cattle breeds reported the Jutland cattle to be genetically unique (Brüniche‐Olsen, Gravlund, & Lorenzen, 2012). Over the past centuries, the breed has experienced a severe decline in population size (Brüniche‐Olsen et al., 2012), and a 2004 estimate indicated an overall *N*_E_ for the Jutland cattle breed of around 40 (http://www.fao.org/dad-is/browse-by-country-and-species/en/), suggesting strong drift and high rates of inbreeding. The low *N*_E_ for the Jutland cattle breed was supported by Pertoldi et al. (2014) who analysed genome‐wide profiles with 50K single nucleotide polymorphism (SNP) markers in the Jutland Kortegaard‐lineage. This native livestock breed thus offers an informative case study of how genome‐wide profiles can inform conservation genetic management of small populations at risk.

### 1.2 Genetic drift and selection in native breeds

Breeders and managers working with small populations at risk are often concerned with the genetic uniqueness of these populations (Ginja, Gama, & Penedo, 2010; Kantanen et al., 2000; Withen, Brüniche‐Olsen, Pedersen, European Cattle Genetic Diversity Consortium, & Gravlund, 2011). This issue is relevant for wild and domestic species, including native livestock breeds where survival has been influenced by local environmental conditions. Genomic profiles from such breeds can advance evolutionary research and conservation by improving our understanding of (i) the role of genetic drift versus selection in contemporary within‐breed lineages, (ii) signatures of selection in native versus commercial breeds and (iii) adaptation to different landscapes in native breeds.

For the Jutland cattle, a long‐standing discussion among farmers and managers in Denmark has been whether lineages should be kept separate or managed as one population. Analyses of genomic profiles from all four contemporary Jutland cattle lineages will thus allow us to determine the amount and distribution of diversity within the breed and among individual lineages. This issue has relevance across livestock breeds where managers acknowledge that rapid conservation actions may be needed to preserve small and declining populations, yet they have concerns that admixture of within‐breed lineages may risk further loss of unique genetic variation, with potential negative implications for locally adapted traits (Hoffmann, 2013; Taberlet et al., 2008). Vital management considerations include the costs and benefits of maintaining separate lineages with few remaining individuals, and preservation of characteristics potentially limiting productivity (e.g., milk, wool, meat) but augmenting survival in harsh environments such as areas with extreme temperatures or precipitation levels, or in habitats with poor‐quality food sources (Hoffmann, 2013; Pariset, Joost, Marsan, & Valentini, 2009).

Preservation of semi‐natural and cultural landscapes has been recognized as a key priority in Europe (Halada, Evans, Romao, & Petersen, 2011; Timmermann, Damgaard, Strand, & Svenning, 2014), and native breeds appear well‐suited to extensive agriculture and biodiversity maintenance in the form of grazing, which can simultaneously offer suitable conditions for in situ natural selection by means of exposure to changing environmental conditions (Hoffmann, 2013). Northern European countries bordering the Atlantic Ocean have cool and humid climates, which may exert selective pressures on domestic species (Pariset et al., 2009). Yet within this region, there are substantial differences in landscape form and terrain ruggedness. In this study, we compare the Jutland cattle from Denmark, dominated by a relatively flat terrain, to native breeds from rugged landscapes in Norway and the Faroe Islands, enabling an investigation of adaption to local conditions (Bailey et al., 2015; Raqiz, Tareen, & Verdier, 2011).

### 1.3 The potential role of cryopreservation in preserving native breeds

The carrying capacity of a population can be increased artificially (i.e., without expanding the in situ population) by supplying genetic material from less related earlier generations of animals. This strategy can be applied to native breeds of cattle and other species by cryopreservation of spermatozoa and oocytes in a gene bank (Curry, 2000; Su et al., 2012). An increasing number of species will, in the near future, be managed with the help of cryopreservation techniques (Charlton et al., 2018), including initiatives such as The Frozen Ark Project (https://www.frozenark.org/). In Denmark, cryopreservation is now used for native livestock breeds including the Jutland cattle, and these collections can provide genetic material for ongoing conservation efforts. Specifically, Hertz et al. (2016) simulated a supplementation in the Jutland Kortegaard‐lineage where one male was added to the population every 5 years, representing cryopreserved semen from previous generations. The simulation suggested that such supplementation can postpone the time to extinction and reduce inbreeding levels (Hertz et al., 2016).

Emerging genomic methods provide exciting prospects for identification of genetic variation and selection in native breeds, yet additional efforts are needed for native breed conservation management to benefit fully from these new approaches (Bruford et al., 2015). The objective of our study was to use within‐breed lineages of the Danish Jutland cattle, native breeds from neighbouring countries that may have been subject to different selective pressures (artificial and natural selection), and commercial dairy breeds selected for high productivity, to investigate (a) the amount and distribution of genetic diversity in a native livestock breed with several lineages, which over recent decades have declined in numbers and are believed to have experienced bottlenecks and extensive genetic drift; (b) the role of genetic drift versus selection in contemporary lineages within native breeds; (c) signatures of selection in native versus commercial breeds; (d) native breed adaptation to various landscapes and environmental conditions (e.g., terrain ruggedness); and (e) whether cryopreserved semen samples conserved over the past decades offer possibilities for infusing native breeds with new variability from past generations.

## 2 MATERIALS AND METHODS

### 2.1 Genetic diversity within and among native breeds

We compared genomes from the Danish Jutland cattle to native breeds from other northern European countries and to commercial breeds originating from northern Europe (Table 1). The Jutland cattle is an indigenous breed that descends from original black and grey pied cattle from the 16th–18th century, and although the Jutland cattle was once widespread in Denmark, it declined following competition and near‐replacement with larger more productive breeds (Brüniche‐Olsen et al., 2012; Kantanen et al., 2000; Sørensen & Nielsen, 2015). In 1949, the breeding association for the Jutland cattle decided to accept Dutch and German black pied cattle (Friesian) into the studbook, and the resulting highly successful crosses formed the Danish black pied cattle that within a decade had replaced almost all original Jutland cattle (Brüniche‐Olsen et al., 2012; Sørensen & Nielsen, 2015). Subsequently, genetic material from Holstein cattle in North America, where this European breed had been imported and further developed, was introduced into the global population of black pied cattle, and this breed constitutes the modern Holstein (also named Holstein‐Friesian) breed (Sørensen & Nielsen, 2015). This process also occurred in Denmark, however; a Friesian population with limited contribution of Holstein genes has been preserved and this lineage is named SDM‐1965 (DAD‐IS, 2017b).

**Table 1:** The populations included in the study, with cattle breed, number of animals sampled, and the estimated number of existing animals per breed at present

Cattle breed	Number sampled	Estimated number in existence	Country
Jutland cattle	386	895 (2016)^a^	Denmark
Kortegaard‐lineage (Jutland)	131		
Oregaard‐lineage (Jutland)	186		
Vesterbølle‐lineage (Jutland)	20		
Westergaard‐lineage (Jutland)	16		
Old bulls pre‐1980^b^	15		
Old bulls post‐1980^c^ (*n* = 14 Jutland)	18		
SDM‐1965	20	212 (2015)^d^	Denmark
Western Norwegian Fjord cattle	21	692 (2015)^e^	Norway
Western Norwegian Red‐polled cattle	19	139 (2015)^e^	Norway
Faroe cattle	8	Circa 40^f^	Faroe Islands
Holstein	9	904,045 (2016)^g^	Denmark^g^
Jersey	9	142,179 (2016)^h^	Denmark^h^

Notes

Where relevant, we have noted where a lineage or subsampled population belongs to the Jutland cattle by adding this name in parentheses. For Jutland cattle lineages Kortegaard and Oregaard, we subsampled *n* = 20 individuals from each group to equalize sample sizes among groups (details in Materials and Methods).

a DAD‐IS (2017a). Numbers within the various Danish Jutland cattle lineages were not available.

b Old bulls pre‐1980 are cryopreserved semen samples of SDM‐1965 included to evaluate temporal changes in genetic diversity and structure.

c Old bulls post‐1980 are cryopreserved semen samples of *n* = 14 Danish Jutland cattle (one sampled in duplicate) and *n* = 4 SDM‐1965.

d DAD‐IS (2017b).

e Sæther and Rehnberg (2016).

f Li et al. (2005). Additionally, DAD‐IS (2017c) reports >1,240 individuals in 1992. One individual sampled in duplicate.

g DAD‐IS (2017d). This report refers to Danish Holstein and thus the Danish population of this international breed originating from The Netherlands.

h DAD‐IS (2017e). This report refers to Danish Jersey and thus the Danish population of this international breed originating from the island of Jersey in the United Kingdom.

To compare genetic diversity, population structure and selection in Jutland cattle with other native breeds, we sampled the SDM‐1965, and native breeds from rugged landscapes in Norway and the Faroe Islands (Table 1) that could exhibit signs of selection for different traits. Additionally, for comparison of native breeds from extensive agriculture with commercial dairy breeds, we sampled Holstein and Jersey cattle that both originate from regions of northern Europe with a cool and humid climate, and have a history of intense selection for high productivity. Within the Jutland breed, we examined the four existing lineages (Figure 1). Moreover, we investigated cryopreserved material of Danish black pied cattle and SDM‐1965, referred to as old bulls. These cryopreserved samples were divided into pre‐1980 and post‐1980 groups to allow temporal comparison of genetic diversity (Table 1), where the samples collected 1960–1980 may be expected to exhibit higher genetic diversity than that of modern samples. The 15 pre‐1980 cryopreserved samples are listed in the studbook as SDM‐1965. For the post‐1980 samples, four are SDM‐1965 and 14 are classified as Jutland cattle (Supporting information Table S1). For contemporary individuals, we collected ear tissue samples from live animals using standard methods with the nextGen tissue sampling unit by AllFlex (http://www.allflexusa.com/, following the manufacturer's recommendations) with the help of cattle owners and managers, and obtained semen samples from Viking Genetics (http://www.vikinggenetics.com/). Samples were genotyped with the Illumina BovineHD array with 776,665 SNPs at Genoskan A/S (Aarhus, Denmark) following the manufacturer's protocol and based on the UMD 3.1 bovine genome assembly. Duplicate samples of two individuals, one old bull post‐1980 and one from the Faroe Islands, were included and allowed comparison of genotyping consistency. The data were filtered in PLINK 1.9 (Purcell et al., 2007) for individual genotyping success of at least 90%, SNP genotyping success of minimum 98% and minor allele frequency (MAF) of 1%. To obtain equalized sample sizes, we then subsampled the more numerous Kortegaard and Oregaard‐lineages by including the first 20 individuals from a list of sample IDs, without any additional knowledge of the individuals, for further analyses. Accordingly, the analyses were performed with reduced sample size for the Kortegaard and Oregaard‐lineages, except for one comparative principal component analysis (PCA) to evaluate the data for these two groups without downsampling (Supporting information Figure S1).

We pruned the data for loci in linkage disequilibrium (LD), and because the Jutland cattle lines have small populations and low genetic diversity where LD is expected to be high, we filtered the data to remove highly linked SNPs. For filtering, we used the PLINK formula (–indep 50 5 2), where 50 is the size of the sliding window (i.e., 50 bp at a time are examined for linked loci), 5 is the number of SNPs shifted in each step and 2 is the variance inflation factor (see further details at http://zzz.bwh.harvard.edu/plink/summary.shtml). We then examined genetic structure in the data with PCA in the *adegenet *package (Jombart, 2008; Jombart & Ahmed, 2011) in R 2.14.2 (R Development CoreTeam, 2012) and with ADMIXTURE (Alexander, Novembre, & Lange, 2009). In ADMIXTURE, the number of population clusters (*K*) is determined with a cross‐validation procedure, where the optimal *K*‐value has the lowest cross‐validation error relative to alternate *K*‐values (Alexander, Shringarpure, Novembre, & Lange, 2015). We examined a range of *K*‐values from 1 to 15 and used 20 cross‐validations for each *K*‐value and 1,000 bootstrap replicates. Subsequently, to provide another measure of differentiation among breeds and lineages, we calculated pairwise *F*_ST_ in Genepop (4.6) (Raymond & Rousset, 1995; Rousset, 2008). For each pairwise comparison, we evaluated the statistical significance of population differentiation by permutations in GenoDive v.2.0b23 (Meirmans & van Tienderen, 2004) with 50,000 randomly selected SNP loci. We applied the Bonferroni correction for multiple comparisons (Rice, 1989). We used TreeMix 1.12 (Pickrell & Pritchard, 2012; http://gensoft.pasteur.fr/docs/treemix/1.12/treemix_manual_10_1_2012.pdf sections 4.1 and 4.4) to build a maximum‐likelihood tree and examine signs of possible admixture among populations. We analysed 195 individuals and 595,025 SNPs pruned for MAF and genotyping success in PLINK, as outlined above, and plotted a maximum‐likelihood tree with the R script plotting_funcs.R provided in the source code for TreeMix (https://bitbucket.org/nygcresearch/treemix/wiki/Home). To measure the amount of genetic diversity within breeds, and within Jutland cattle lineages, we calculated polymorphism (P%), observed (*H*_O_) and expected heterozygosity (*H*_E_) with standard error, and pairwise identity by descent (IBD) between individuals within breeds/lineages in PLINK. Furthermore, we calculated *N*_E_ with 95% confidence intervals in NeEstimator v2.1 (Do et al., 2014) with the LD method (Waples & Do, 2008) and the data set pruned for LD. The same data set was used to calculate the number of private alleles (*P*_A_) per population in R with a script (https://johnbhorne.wordpress.com/2017/07/12/identifying-private-snps-in-r/) for the HierfStat package (Goudet, 2005).

### 2.2 Genetic drift and selection in native breeds

To evaluate whether genomic profiles of different breeds showed signs of selection, we performed tests of outlier loci in the *pcadapt* package (Luu, Bazin, & Blum, 2017) in R. The program incorporates a false discovery rate (FDR) approach to account for multiple testing that permits users to select a specific alpha‐level (Luu et al., 2017, https://bcm-uga.github.io/pcadapt/articles/pcadapt.html). We used equalized population sizes and an alpha‐level (*q*‐value) of 0.01 for detecting loci that deviated significantly from the neutral distribution, whereby loci with *q*‐values <0.01 were considered outliers. We performed seven separate tests for outlier loci (Table 2). These included six tests for signs of possible selection linked to local environmental conditions and one comparison of two Jutland cattle lineages that show signs of genetic structure believed to have been produced by genetic drift. Although the latter test does not represent any true control, we intended it to provide context towards understanding how the outlier test performs in the event of (presumed) genetic drift only. Firstly, T1 examined all population clusters discovered (*K* = 9; see Results). The subsequent T2–T7 were pairwise tests, each time with two relevant populations, to help identify unique variants or results shared across two or more populations (Table 2). The T2 tested the Kortegaard versus the Oregaard‐lineages of Jutland cattle, two relatively recent lineages where observed genetic divergence (Brüniche‐Olsen et al., 2012) is expected to be based on drift with no known differences in selection regimes. Subsequently, T3 compared 1960–1980 cryopreserved Danish SDM‐1965 with Holstein cattle (test of native vs. commercial breeds), T4 tested 1960–1980 cryopreserved Danish SDM‐1965 versus Western Norwegian Red‐polled cattle (comparing two native breeds), and T5 compared the Western Norwegian Red‐polled cattle with Western Norwegian Fjord cattle (comparing two native breeds, where the Western Norwegian Red‐polled cattle are larger and polled, i.e., without horns). Finally, T6 compared Jutland cattle, represented by old Jutland bulls post‐1980, with Holstein cattle (test of native vs. commercial breeds), and T7 tested the old Jutland bulls post‐1980 versus Western Norwegian Red‐polled cattle (comparing two native breeds). For brevity, we subsequently refer to these tests as T1–T7 (Table 2).

**Table 2:** Tests for the presence of outlier loci in cattle breeds, including one test across all breeds (T1), and one test used as a measure of control (T2) where differentiation is expected to be explained by recent genetic drift without any known history of selection

Test	Cattle included	Test type	Performed to evaluate
T1	All	Across all groups (*n* = 9)	Outliers among all groups
T2	Jutland Kortegaard versus Oregaard‐lineages	Pairwise	Genetic drift in native breed lineages (few/no results expected)
T3	Old bulls pre‐1980 (cryopreserved SDM‐1965) versus Holstein cattle	Pairwise	Native versus commercial breed; selection for local environmental conditions in native cattle
T4	Old bulls pre‐1980 (cryopreserved SDM‐1965) versus Western Norwegian Red‐polled cattle	Pairwise	Two native breeds; selection for different landscape types (rugged terrain in Western Norway, gentle terrain in Denmark)
T5	Western Norwegian Red‐polled cattle versus Western Norwegian Fjord cattle	Pairwise	Two native breeds; selection for polled phenotype (without horns) in Red‐polled cattle
T6	Old bulls post‐1980 (cryopreserved Jutland) versus Holstein cattle	Pairwise	Native versus commercial breed; selection for local environmental conditions in native cattle
T7	Old bulls post‐1980 (cryopreserved Jutland) versus Western Norwegian Red‐polled cattle	Pairwise	Two native breeds; selection for different landscape types (rugged terrain in Western Norway, gentle terrain in Denmark)

Note

A description of cattle breeds and lineages is provided in Table 1.

For SNPs identified as outliers under divergent selection, we screened the flanking region of the bovine genome in the NCBI Map Viewer, annotation release 104 (https://www.ncbi.nlm.nih.gov/projects/mapview/map_search.cgi?taxid=9913). We followed the approach in Porto‐Neto et al. (2014) in their study of climate adaptation in cattle and examined 3,000 base pairs (bp) on each side of outlier SNPs to identify functional genes under known or potential selection. We noted genes found within the 3,000‐bp flanking regions of the outliers and examined the genes with the NCBI Gene website (https://www.ncbi.nlm.nih.gov/gene/) for information on gene function in cattle and other species (primarily records from *Homo sapiens* and *Mus musculus*). We centred on genes relevant to selection in cattle and our investigation of native breeds (see Supporting information Table S2 for a list of all genes) and chose genes for in‐depth investigation (henceforth focal genes) using the bibliography from NCBI Gene and additional references obtained from literature searches.

For focal genes, we examined possible enrichment in gProfiler (Reimand et al., 2016) with the Gene Ontology (GO) Biological Processes and Human Phenotype Ontology databases (https://biit.cs.ut.ee/gprofiler/index.cgi). We followed the approach of Caniglia et al. (2018) and limited the size of functional categories to maximum 500 terms, to focus our analyses on more specific genome regions. We chose the gProfiler g:SCS significance threshold for multiple testing as recommended by the program authors (Reimand et al., 2016).

We examined and compared runs of homozygosity (ROH) in PLINK for representative groups including two native breeds from different environments and one commercial breed. These were the native Jutland cattle (old bulls post‐1980), the native Western Norwegian Red‐polled cattle and the commercial Holstein breed. ROH represent long homozygous segments in individuals where both parents have transmitted identical haplotypes, and can indicate various processes that include selection and recent inbreeding (Purfield, Berry, McParland, & Bradley, 2012). We used data for autosomal loci filtered for MAF and genotyping success, and PLINK functions —homozyg and —homozyg‐group with default parameters. These parameters permit one heterozygote and five missing genotypes within windows of 5 Mb and 50 SNPs. To compare results from different methods to detect possible selection, we plotted the distribution of focal genes detected in outlier flanking regions and ROH across all autosomal chromosomes based on the approach in Stronen et al. (2017), comparing Jutland versus Holstein cattle, and Jutland versus Western Norwegian Red‐polled cattle.

## 3 RESULTS

### 3.1 Genetic diversity within and among native breeds

**Figure 2:**
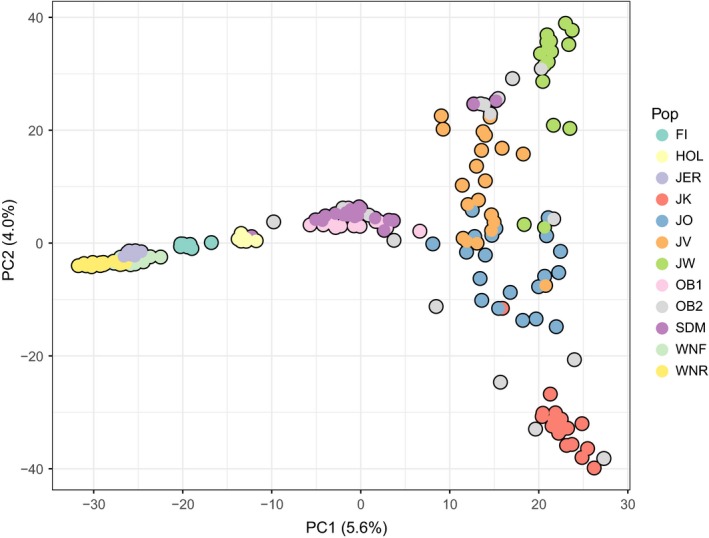
Principal component analyses (PCAs) with 195 individuals showing the first and second PC axes. Cattle breeds/lineages (denoted in figure legend as Pop) are as follows: FI: Faroe Island cattle; HOL: Holstein; JER: Jersey; JK: Jutland cattle Kortegaard‐lineage; JO: Jutland cattle Oregaard‐lineage; JV: Jutland cattle Vesterbølle‐lineage; JW: Jutland cattle Westergaard‐lineage; OB1: old bulls pre‐1980 (cryopreserved semen samples from SDM‐1965 cattle); OB2: old bulls post‐1980 (cryopreserved from *n* = 14 Jutland and *n* = 4 SDM‐1965 cattle); SDM: SDM‐1965 cattle; WNF: Western Norwegian Fjord cattle; WNR: Western Norwegian Red‐polled cattle

Quality screening of the data produced 710,471 SNPs. After equalizing sample sizes (Table 1), we obtained a final sample of 195 individuals and 677,311 autosomal SNPs filtered for genotyping success (as defined above per individual and per SNP), and 88,190 SNPs after linkage pruning. PCA plots (Figure 2, Supporting information Figure S2) showed Norwegian and Faroe Island cattle, commercial breeds, old bulls, SDM‐1965 and contemporary Jutland cattle lineages distributed along PC1. The four contemporary Jutland lineages were divergent from old bulls pre‐1980 and most SDM‐1965 individuals on PC1, with the more recent samples from old bulls post‐1980 broadly scattered. PC2 showed separation among Jutland cattle lineages, where the Kortegaard‐ and Westergaard‐lineages emerged as most divergent. The third PC separated old bulls pre‐1980 and SDM‐1965; PC4 highlighted the Jutland Westergaard‐lineage; and PC5 highlighted differentiation between Western Norwegian Red‐polled and Jersey cattle (Supporting Information Figure S2).

**Figure 3:**
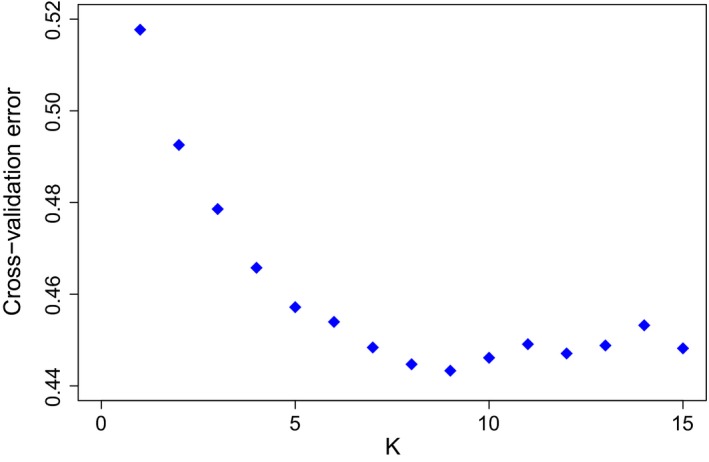
ADMIXTURE analyses of cattle with cross‐validation (CV) error plot for *K*‐values from 2 to 9 with 195 individuals and 88,190 single nucleotide polymorphism loci. The CV error is markedly reduced with each increase in *K* until *K* = 5. Hereafter it declines more slowly towards the minimal value at *K* = 9. Increases in *K* beyond this level are not supported

**Figure 4:**
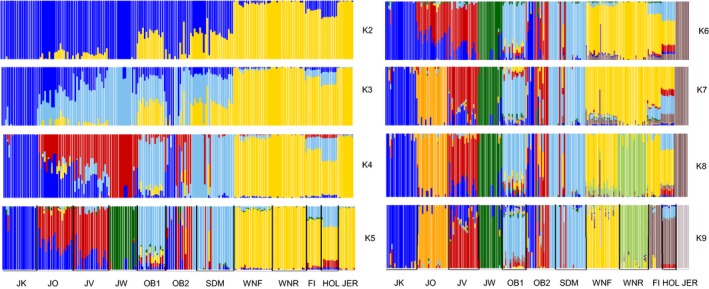
ADMIXTURE plots for *K*‐values from 2 to 9 clusters with 195 individuals and 88,190 single nucleotide polymorphism loci. Each vertical bar represents one individual, and the Y‐axis shows individual ancestry (range: 0–1). Cattle breeds/lineages are as follows: FI: Faroe Island cattle; HOL: Holstein; JER: Jersey; JK: Jutland cattle Kortegaard‐lineage; JO: Jutland cattle Oregaard‐lineage; JV: Jutland cattle Vesterbølle‐lineage; JW: Jutland cattle Westergaard‐lineage; OB1: old bulls pre‐1980 (cryopreserved semen samples from SDM‐1965 cattle); OB2: old bulls post‐1980 (cryopreserved from *n* = 14 Jutland and *n* = 4 SDM‐1965 cattle); SDM: SDM‐1965 cattle; WNF: Western Norwegian Fjord cattle; WNR: Western Norwegian Red‐polled cattle

The ADMIXTURE results showed the highest support for *K* = 9, although it should be noted that the rate of reduction in the CV error showed a marked decline at *K* = 5 (Figure 3). The ADMIXTURE results for *K* = 2 exhibited differentiation between Danish native cattle and other breeds. New units that emerged at *K*‐values of 3–5 were the Westergaard‐lineage, the Kortegaard‐lineage, the old bulls pre‐1980 (i.e., SDM‐1965) and modern SDM‐1965 (Figure 4). The *K* = 4 value separated Danish native cattle, that is, the four Jutland cattle lineages and SDM‐1965 with Jutland ancestry, from all other commercial and foreign breeds (for a detailed discussion of Jutland lineages, see Supporting Information Appendix S1). At *K* = 5, the Westergaard‐lineage constituted a separate group within the Jutland cattle whereas the Kortegaard‐lineage and a portion of the old bulls post‐1980 were assigned to one cluster, to which individuals from the Oregaard‐lineage and the Vesterbølle‐lineage also showed partial affinity. SDM‐1965, old bulls post‐1980 and some of the old bulls pre‐1980 formed another cluster. Other non‐Danish breeds emerged with subsequent increases in *K*‐values. The results for *K* = 9 provided additional resolution including differentiation among the four Jutland cattle lineages, with individuals from the Vesterbølle‐lineage showing partial membership in the cluster representing the Kortegaard‐lineage. Modern SDM‐1965 and the old bulls pre‐1980 samples of SDM‐1965 showed substantial overlap, whereas the old bulls post‐1980 emerged as a highly admixed group with representation primarily in the Kortegaard‐ and Vesterbølle‐lineages. Each of the Norwegian breeds and the Jersey cattle appeared as separate clusters, and there was no indication of introgression into the Jutland cattle. In contrast, there was no obvious distinction in ADMIXTURE between the Faroe Island and Holstein individuals. The two individuals with duplicate samples showed consistent genomic profiles.

**Figure 5:**
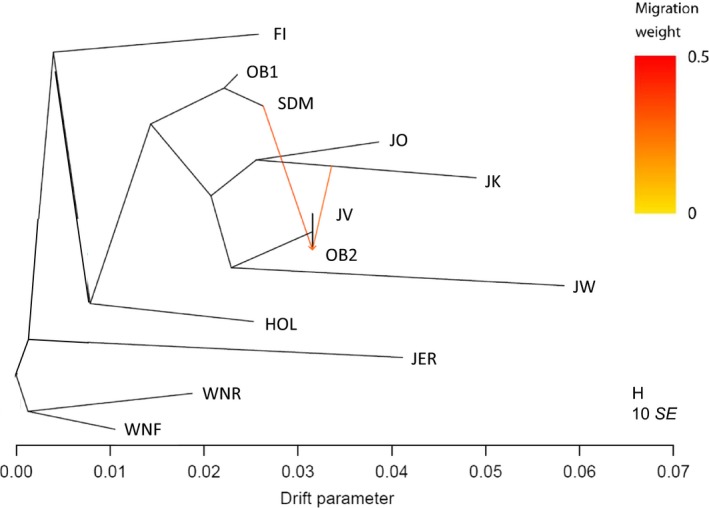
Maximum‐likelihood tree for 12 cattle populations with 195 individuals and 595,025 SNPs pruned for minor allele frequency of 1% and genotyping success of 98%. The scale bar on the horizontal axis shows 10× the average standard error of the sample covariance matrix, and the length of horizontal branches is proportional to the amount of genetic drift the populations have experienced. Cattle breeds/lineages are as follows: FI: Faroe Island cattle; HOL: Holstein; JER: Jersey; JK: Jutland cattle Kortegaard‐lineage; JO: Jutland cattle Oregaard‐lineage; JV: Jutland cattle Vesterbølle‐lineage; JW: Jutland cattle Westergaard‐lineage; OB1: old bulls pre‐1980 (cryopreserved semen samples from SDM‐1965 cattle); OB2: old bulls post‐1980 (cryopreserved from *n* = 14 Jutland and *n* = 4 SDM‐1965 cattle); SDM: SDM‐1965 cattle; WNF: Western Norwegian Fjord cattle; WNR: Western Norwegian Red‐polled cattle. Migration arrows are coloured according to their weight and indicate admixture between old bulls post‐1980 and Kortegaard, as well as between old bulls post‐1980 and SDM‐1965

Genetic differentiation measured by pairwise *F*_ST_ values varied between native and commercial breeds, and between the native breeds and lineages (Table 3). For Jutland cattle, pairwise comparisons between lineages all showed *F*_ST _>0.1 except for Oregaard–Vesterbølle. Moreover, pairwise comparison with Norwegian breeds and Faroe cattle all showed *F*_ST _>0.1 and suggested clearly differentiated populations. Although ADMIXTURE did not separate Faroe Island and Holstein cattle, the pairwise *F*_ST_ values of 0.1131 showed some differentiation. The TreeMix results indicated that the old bulls post‐1980 had experienced admixture with the Kortegaard‐lineage and with SDM‐1965 (Figure 5). Analyses of genetic drift between native Jutland Kortegaard and Oregaard‐lineages were supported by PCA (Figure 2, Supporting Information Figures S1 and S2), ADMIXTURE (Figure 4) and the *F*_ST_ value of 0.1034. Among Jutland cattle lineages, Westergaard was shown to have experienced the highest level of genetic drift, followed by Kortegaard.

**Table 3:** Estimates of genetic differentiation calculated as *F*_ST_ for all pairs of cattle breeds/lineages

Breed/lineage	Kortegaard (*n* = 20)	Oregaard (*n* = 20)	Vesterbølle (*n* = 20)	Westergaard (*n* = 16)	Old bulls pre‐1980 (*n* = 15)	Old bulls post‐1980 (*n*=14^a^)	SDM‐1965 (*n* = 20)	Vestlandsk Fjordfe (*n* = 21)	Vestlandsk Raudkolle (*n* = 19)	Faroe cattle (*n* = 8)	Danish Holstein (*n* = 9)
Jutland Oregaard‐lineage (*n* = 20)	0.1034	–									
Jutland Vesterbølle‐lineage (*n* = 20)	0.1047	0.0780	–								
Jutland Westergaard‐lineage (*n* = 16)	0.1822	0.1457	0.1238	–							
Old bulls pre‐1980 (*n* = 15)	0.1121	0.0959	0.0709	0.1403	–						
Old bulls post‐1980 (*n*=14^a^)	0.0609	0.0487	−0.0009^1^	0.1046	0.0486	–					
SDM‐1965 (*n* = 20)	0.1192	0.0995	0.0701	0.1403	0.0163^2^	0.0491	–				
Western Norwegian Fjord cattle (*n* = 21)	0.1371	0.1159	0.1016	0.1618	0.0833	0.0801	0.0919	–			
Western Norwegian Red‐polled cattle (*n* = 19)	0.1589	0.1358	0.1217	0.1838	0.1048	0.1007	0.1125	0.0713	–		
Faroe cattle (*n* = 8)	0.1742	0.1488	0.1301	0.2030	0.1061	0.1069	0.1148	0.0849	0.1142	–	
Holstein (*n* = 9)	0.1565	0.1326	0.1096	0.1866	0.0872	0.0866	0.0898	0.0900	0.1116	0.1131	–
Jersey (*n* = 9)	0.2180	0.1912	0.1749	0.2474	0.1547	0.1529	0.1614	0.1242	0.1524	0.1711	0.1578^3^

Notes

All pairwise comparisons were significant at *p* < 0.001, except the values marked with 1 (*p* = 0.463), 2 (*p* = 0.004) and 3 (*p* = 0.002) after overall Bonferroni correction for multiple comparisons (Rice, 1989) (*K* = 66).

a Including only cryopreserved semen samples of *n* = 14 Danish Jutland cattle recognized in the studbook.

Results for *H*_O_ and *H*_E_ showed clear differences among breeds and lineages, also within the Jutland breed (Table 4). Genetic diversity was generally lower for Jutland cattle than for other groups, with the lowest values observed for the Westergaard‐lineage. In contrast, old bulls pre‐1980 had some of the highest values observed. Although the Holstein, Jersey and Faroe cattle had the lowest numbers of individuals tested, the Holstein and Faroe cattle showed comparatively high *H*_E_ and *H*_O_ values. Polymorphism also varied among populations, with the lowest value (67.8%) found in the Westergaard‐lineage and the highest value (84.1%) observed in the Western Norwegian Fjord cattle (Table 4). Values for IBD differed among populations and in general showed an inverse pattern to that of genetic diversity, with the highest mean value observed in the Westergaard‐lineage and the lowest mean value in the old bulls post‐1980 followed by the Western Norwegian Fjord cattle (Table 4, Supporting Information Table S3 and Supporting Information Figure S3). The results for *N*_E_ exhibited relatively broad variation with the smallest value found for the Westergaard‐lineage and the highest estimate observed in the Holstein cattle (Table 4). The values for *P*_A_ ranged from none in the old bulls post‐1980 to 221 being found in the Western Norwegian Red‐polled cattle (Table 4). Within Jutland lineages, Kortegaard had the lowest number (*n* = 2) and Oregaard the highest (*n* = 32), with intermediate values for Vesterbølle (*n* = 5) and Westergaard (*n* = 9).

**Table 4:** The number of samples analysed (No), observed (*H*_O_) and expected heterozygosity (*H*_E_) values with standard error (*SE*), per cent polymorphic loci (P%), identity by descent (IBD) per cattle group, effective population size with 95% confidence interval (*N*_E_), and number of private alleles (*P*_A_)

Cattle breed/lineage	No	*H*_O_ (*SE*)	*H*_E_ (*SE*)	P%	IBD mean (range)	*N*_E_	*P*_A_
Jutland Kortegaard‐lineage	20	0.2618 (0.00026)	0.2474 (0.00023)	73.1	0.296 (0–0.642)	9.1 (9.1–9.2)	2
Jutland Oregaard‐lineage	20	0.2776 (0.00026)	0.2647 (0.00023)	75.8	0.261 (0–0.677)	10.0 (10.0–10.0)	32
Jutland Vesterbølle‐lineage	20	0.2833 (0.00026)	0.2752 (0.00022)	78.8	0.218 (0–0.620)	21.6 (21.5–21.7)	5
Jutland Westergaard‐lineage	16	0.2532 (0.00028)	0.2267 (0.00023)	67.8	0.384 (0.187–0.724)	4.0 (4.0–4.1)	9
Old bulls pre‐1980	15	0.3034 (0.00026)	0.2860 (0.00022)	81.3	0.216 (0–0.591)	24.8 (24.7–24.9)	7
Old bulls post‐1980^a^	14	0.2800 (0.00024)	0.2857 (0.00022)	80.7	0.134 (0–0.685)	11.8 (11.7–11.8)	–
SDM‐1965	20	0.2842 (0.00024)	0.2838 (0.00022)	81.2	0.160 (0–0.592)	18.0 (17.9–18.0)	9
Western Norwegian Fjord cattle	21	0.3035 (0.00024)	0.2983 (0.00021)	84.1	0.151 (0–0.566)	35.0 (34.9–35.1)	216
Western Norwegian Red‐polled cattle	19	0.2980 (0.00025)	0.2849 (0.00022)	80.6	0.216 (0–0.609)	18.0 (18.0–18.1)	221
Faroe cattle	8	0.2997 (0.00029)	0.2707 (0.00023)	75.0	0.293 (0.196–0.646)	12.2 (12.2–12.3)	55
Holstein	9	0.3053 (0.00028)	0.2836 (0.00022)	77.0	0.241 (0.209–0.341)	92.0 (89.6–94.4)	45
Jersey	9	0.2705 (0.00029)	0.2483 (0.00023)	69.9	0.327 (0.272–0.506)	28.9 (28.6–29.2)	112

Notes

N_E_ and P_A_ were calculated with LD‐pruned data. Further IBD details are provided in Supporting Information Table S3 and Supporting Information Figure S3.

a Including only cryopreserved semen samples of *n* = 14 Danish Jutland cattle recognized in the studbook.

### 3.2 Genetic drift and selection in native breeds

Analyses of SNPs under potential selection identified 1985 loci, some of which were found in multiple tests (Supporting Information Table S2). The number of observed outliers differed among tests, with T1 producing *n* = 229 loci, T2: *n* = 62, T3: *n* = 336, T4: *n* = 199, T5: *n* = 235, T6: *n* = 425 and T7: *n* = 766 outliers. After examination of all outliers (Supporting Information Table S2), we selected *n* = 146 focal genes and genome regions for further examination based on their relevance for livestock and native breeds (Table 5, Supporting Information Appendix S2). We categorized these findings as production traits (growth and meat quality, *n* = 46 genes), milk production (*n* = 11), reproduction (*n* = 35), physical appearance (*n* = 2), climate adaptation (*n* = 6), behaviour and cognition (*n* = 10), hormones (*n* = 2), infection and immunity (*n* = 11), metabolism (*n* = 15), and sensory including olfaction (*n* = 3), vision (*n* = 3) and hearing (*n* = 2; Table 5, Supporting Information Appendix S2, Supporting Information Table S4), and these traits may be influenced by artificial and/or natural selection.

**Table 5:** Focal genes found in the 3,000‐bp flanking regions of outlier single nucleotide polymorphism loci in native and commercial cattle

Category	T1 (all cattle breeds/lineages)		T2 (Jutland lineages Kortegaard vs. Oregaard)	T3 (1960–1980 SDM‐1965 vs. Holstein)	T4 (1960–1980 SDM‐1965 versus Western Norwegian Red‐polled			T5 (Western Norwegian Fjord versus Red‐polled	T6 (post‐1980 Jutland vs. Holstein)	T7 (post‐1980 Jutland versus Western Norwegian Red‐polled cattle	
Production traits (growth, meat)	*ME1, TRPV4, LRP2*		*IGF1*	*LRP2, CTTNBP2NL, MECOM, PTPN1*	*PRDM16, ADAMTSL3*			*FLT1*	*ARHGEF3, BMP7, CEP128, CYP2J2, ERCC6L2, ESRRG, FBN1, FGD3, FLVCR1, IARS, MYOM3, PALM2, PHLDB2, ROR2, SLC8A1, ZNFX1*	*AKT3, BBX, BMP7, COL5A1, DCHS2, DIS3L2, DSCAM, ESRRG, FAP, FBLN7, FBN1, FGD3, FLVCR1, F10, GLRA1, GUCY1A1, HPS5, IARS, MYBPC1, PDE1A, PHLDB2, RIN3, ROR2, RPS6KA4, RUNX2, SMURF1, TENM4, TTN*	
Milk production	*BTC, ITGA6, PDE4D*		*IGF1*	*ABCG2, CTNND1, TCF7L2, CUX1*		*SLC2A8/GLUT1, ATP1A1*	*PDE4D*		*ITGA6*	*ITGA6, SLC5A1*	
Reproduction	*ITGA6, HSD17B12, SOX5, DPH6/ATPBD4, LRP2, BIRC5*	*IGF1*		*LRP2, GABRA4, LHX6, LRRC34, NR3C1, ABCG2, CTTNBP2NL, RXFP1/LGR7*		*ELMO1*	*BIRC5*		*AVEN, CD9, EBF1, GSTM1,* *SPEF2, NPHP4, SPATA31A3, STAU2, UNC5C, ZNF462*		*ATP6V0A2, CATSPERD, HOOK1, NRG1, NTRK2, PATE1, RANBP9, SPATA31A3, UBE2E3, WSB2, ZP2*
Physical appearance						*KRT31*	*KRT31*				*KRTAP*
Climate adaptation	*NUDCD3*			*HSPBP1, SLC18A1/VMAT‐1*		*SLC18A1/VMAT‐1, ATP1A1, RPTOR*					*ACBD6*
Behaviour and cognition	*GRM7, CLSTN2, LSAMP*			*CERS6, GRID2/LOC536367*		*CHRNB2*	*LSAMP*				*DAB1, DSCAM, NETO1, TMEM132D*
Hormones	*SLC26A4*			*SAFB2*							
Infection and immunity	*DMKN, TRAPPC9*			*TRAPPC9, NFATC2IP*		*TRAPPC9, SPAG11B*			*CPNE3, (Ig)M/CADM2, PLCXD3, SIGIRR,*		*CPNE3, (Ig)M/CADM2, GZMB, STXBP6, PRODH2*
Metabolism	*RORA, BTC, GAPDHS*			*LIPF/HGL, PANK1*			*OXCT1*		*EBF1, HACE1, KLHL32,*		*ABCA1, HACE1, KSR2, LRRC8C, NEGR1, RGS5, SDCCAG8*
Sensory—olfaction	*LOC783998/OR11G2*					*NDUFA10*	*NDUFA10*				*LOC783998/OR11G2*/*OR11A1*
Sensory—vision									*CTNND2, NPHP4*		*CTNND2, TENM3*
Sensory—hearing											*PCDH15, TSPEAR*

Notes

Details on tests T1–T7 and populations are given in Table 2; gene information and references are provided in Supporting Information Appendix S2 and Supporting Information Table S4.

**Figure 6:**
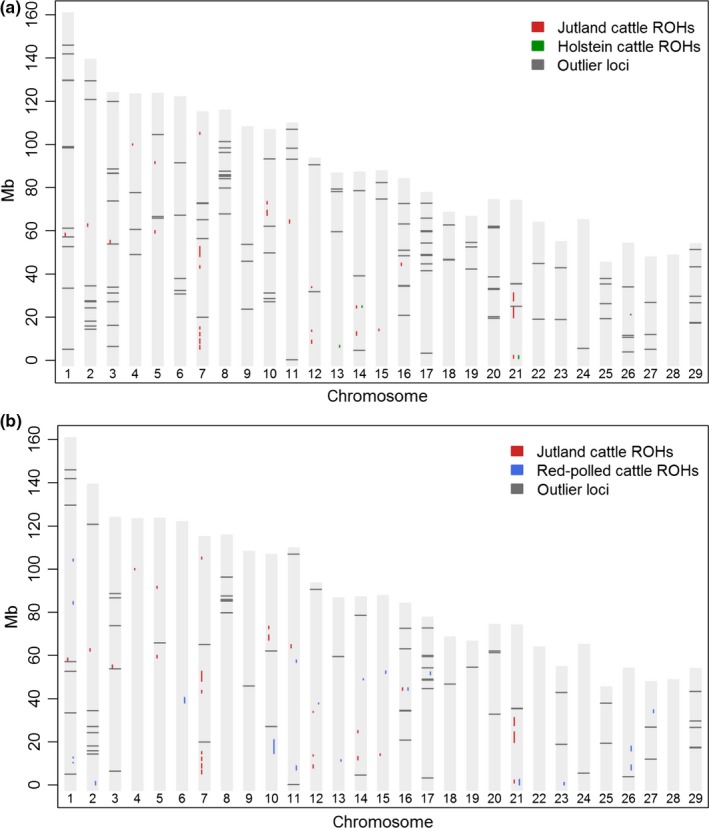
Plots displaying runs of homozygosity (ROH) per autosomal chromosome and outliers from pairwise comparisons of native and commercial breeds central to this study. Outliers and ROH could both be indicative of selection, and we mapped our findings to examine the degree of overlap. ROH shared by at least six individuals per breed are shown in vertical coloured lines. Outlier loci where focal genes were found within 3,000‐bp flanking regions are marked as dark grey horizontal lines. The plots show pairwise comparisons of (a) Jutland cattle versus Holstein cattle, and (b) Jutland cattle versus Western Norwegian Red‐polled cattle

Comparisons of the outlier results show that the majority of genes identified were associated with production traits (growth/meat) and reproduction. The distribution of results was uneven, ranging from T2 where we identified one flanking gene (*IGF1*) linked to production traits, milk and reproduction to T7 where we detected numerous genes associated with production traits (*n* = 27), reproduction (*n* = 11) and other traits (Table 5). Genes linked to milk production were identified in all comparisons, with the majority identified in T3 (*n* = 4). In contrast, genes associated with physical appearance were found only in comparisons involving the Western Norwegian Red‐polled cattle, and all results were related to the polled phenotype exhibited by this breed. Enrichment analyses identified GO categories (*n* = 19) for production traits that included cellular response to growth factors, muscle development and bone formation (Supporting Information Table S5), and for milk, we observed categories (*n* = 6) primarily linked to transmembrane transport. For behaviour and cognition, we identified GO categories (*n* = 11) including locomotory behaviour and synaptic signalling (Supporting Information Table S5), whereas for hearing, we detected categories (*n* = 3) for sensory perception of sound and mechanical stimulus, including equilibrioception (the sense of balance). Genes linked to other types of traits produced no significant GO results. Examination of ROH found that none were shared among all individuals within breeds. We plotted ROH shared among at least six individuals per breed, and focal genes found within outlier flanking regions, across all autosomal chromosomes (Figure 6a,b).

### 3.3 The potential role of cryopreservation in preserving native breeds

Cryopreserved samples exhibited high genetic diversity, with seven *P*_A_ found in the pre‐1980 sample of SDM‐1965 cattle (Table 4). As expected, we also observed higher genetic variability in the 15 pre‐1980 samples of SDM‐1965 cattle (*H*_O_ = 0.3034, *H*_E_ = 0.2860) than in the 20 modern samples of SDM‐1965 (*H*_O_ = 0.2842, *H*_E_ = 0.2838). Mean IBD was also higher (0.216 vs. 0.160) and P% very similar (81.3 vs. 81.2). The post‐1980 samples of Jutland cattle did not exhibit *P*_A_, but the 14 sampled bulls showed higher *H*_E_ and lower IBD than all contemporary Jutland lineages, and higher *H*_O_ than all but one of them (the Vesterbølle‐lineage; Table 4).

## 4 DISCUSSION

Our genome‐wide study of the amount and distribution of genetic diversity in native breeds documented distinct native and commercial cattle breeds. The Jutland cattle exhibited clear substructure, where increased *N*_E_ may be needed for long‐term conservation management, particularly given the reduced genetic diversity found within the lineages with the lowest census population sizes. Comparison of commercial and native breeds identified genes in the native breeds that merit further study concerning trade‐offs between commercial output versus animal health, robustness in low‐input habitats, and adaptation to changing environments, such as immune function and metabolism. Native breeds may also comprise important genetic diversity for local adaptive variation, including heat tolerance, spatial learning and memory. Importantly, cryopreserved samples exhibit high and unique genetic variation and present opportunities to increase variation and restore diversity from earlier generations. Finally, we demonstrated how genomics can help towards solving questions of broad interest within conservation and evolutionary genetics that also have practical relevance for breed managers.

### 4.1 Genetic diversity within and among native breeds

Our results suggest the Danish Jutland cattle offer an informative case study on the threats facing many native breeds around the world (FAO, 2015), and the resources these breeds represent for long‐term preservation of genetic variation in domestic animals (Hoffmann, 2013). Our findings on genetic structure and diversity agree with earlier results (Brüniche‐Olsen et al., 2012) showing Jutland cattle as a distinct breed (see also Supporting Information Appendix S1). Although earlier microsatellite analyses produced mixed results for the Kortegaard‐lineage (Brüniche‐Olsen et al., 2012), our study identified all four contemporary lineages as separate population clusters. The Jutland cattle exhibited genetic similarities to SDM‐1965, as expected from shared ancestry. The two Norwegian native breeds had genetic profiles and diversity that appear consistent with their different phenotypes and history (Kantanen et al., 2000), whereas Faroe Island cattle and Holstein did not emerge as unequivocally distinct genetic units in our study. The genetic background of the Faroe Island cattle is complex (Li, Sternbauer, Haahr, & Kantanen, 2005), and further genome‐wide analyses with a broader range of breeds may be needed to resolve their history. Jutland cattle genetic diversity seems consistent with earlier microsatellite data suggesting comparatively high variability (Brüniche‐Olsen et al., 2012), and our findings of reduced *H*_E_, *H*_O _and polymorphism for the Westergaard‐lineage are concurrent with the lower levels of mtDNA haplotype variation (Brüniche‐Olsen et al., 2012) found for this lineage. However, a direct comparison between the two studies is not possible and differences could, at least in part, be explained by the properties of different marker types with microsatellites typically exhibiting rapid mutation rates and a bias towards highly polymorphic loci (Brandström & Ellegren, 2008). An earlier study based on data from a lower‐density chip with 50K SNPs reported lower genetic diversity in the Kortegaard‐lineage than for a range of commercial breeds (Pertoldi et al., 2014). Moreover, Pertoldi et al. (2014) reported *H*_O_ = 0.266 and *H*_E_ = 0.259, and we observed 0.262 and 0.247, respectively; thus, similar levels of heterozygosity were found in the two studies.

Within the Jutland cattle, we observed the highest IBD values for the Westergaard‐lineage, followed by Kortegaard, Oregaard and Vesterbølle, whereas Brüniche‐Olsen et al. (2012) found strong signatures of inbreeding (significant deviation from Hardy–Weinberg equilibrium due to heterozygosity deficiency) for all groups except Oregaard. Inclusion of different marker types, sample sizes and individuals may have contributed to discrepancies among studies (Supporting Information Appendix S1). However, the range of observed IBD values suggests there are opportunities to carefully select individuals to minimize future inbreeding in ongoing conservation management. Additional considerations for selection of breeding individuals are long‐term conservation of SNPs under potential selection, and preservation of such variants may be considered a key aim of breeding strategies for native breeds. We observed relatively low polymorphism (67.8%) and high IBD (0.384) in the 16 Westergaard individuals compared to those found in nine Danish Holstein cattle (69.9% and 0.327, respectively). However, in a broader perspective, the polymorphism and IBD values for the investigated native breeds suggest these comprise important diversity relative to their considerably smaller census population sizes than those of commercial breeds (Table 1). Altogether 1,019 native cattle breeds have been reported globally, with 369 in Europe and the Caucasus (FAO, 2015). Understanding and preserving the genetic diversity of native breeds, especially those with small population sizes, is thus a priority, including assessments of epigenetic processes linked to environmental factors (e.g., diet) (FAO, 2015). Adaptation to changing environmental conditions will become increasingly important (Hoffmann, 2013), and the existing diversity within the many native cattle breeds could help facilitate more rapid adaptation.

Diverse patterns of variation may emerge from the use of different markers (Brandström & Ellegren, 2008), but we believe the detailed SNP profiles to be representative for contemporary Jutland cattle breed lineages and to reflect genome‐wide variability and structure within and among breeds in our study. Overall, the results from this and earlier research on Jutland cattle indicate a distinct breed with multiple lineages. Moreover, the *P*_A_ values suggest that considerable variation may be found in smaller populations of native breeds. The old bulls post‐1980 (Jutland bulls) was the only group that did not exhibit any *P*_A_. This is likely explained by its variants being shared among contemporary lineages, which seems consistent with the TreeMix results of admixture involving this group. In contrast, the old bulls pre‐1980 (SDM‐1965) exhibited seven *P*_A_, which seems consistent with the group's high levels of heterozygosity. The two Norwegian breeds also showed a high number of *P*_A_, despite modest values for *N*_E_. The small remaining populations of Vesterbølle and Westergaard, which represent the upper and lower *N*_E_ estimates for Jutland lineages (i.e., subpopulations) with four and 21 individuals, respectively, illustrate the difficulties inherent in managing such lineages as separate units. As suggested for isolated populations of wild species with small *N*_E _(Laikre, Olsson, Jansson, Hössjer, & Ryman, 2016), long‐term management of native livestock breeds might benefit from a metapopulation approach where lineages are considered as metapopulations. Earlier simulations have also suggested that augmenting the breeding pool with individuals of both sexes could help increase the probability of persistence (Hertz et al., 2016).

### 4.2 Genetic drift and selection in native breeds

Our analyses of outliers indicated that the breeds have evolved in consequence of both artificial and natural selection. The relatively high number of genes found associated with production traits and reproduction is likely influenced by the importance of these features for commercial breeds and thus for development of the SNP panel used in our study. The enrichment results also seem to suggest strong research focus on genomic regions important for livestock production (meat and milk) and potentially also for other areas within biology and evolution (hearing, behaviour and cognition). The overall distribution of results among outlier tests appears to support our expectations of broad genome‐wide differences among the investigated populations and the evolutionary uniqueness of native breeds. Danish native cattle exhibited differentiation from commercial breeds, which has also been reported from other regions (Iso‐Touru et al., 2016; Kawahara‐Miki et al., 2011; Lim et al., 2016). Pairwise tests between native breeds also showed signs of divergent selection, which is in accordance with findings from other parts of the world (Iso‐Touru et al., 2016; Stucki et al., 2017). Certain characteristics, such as olfaction (McRae et al., 2013), hormonal cues concerning reproduction (Jiang et al., 2006), and cognitive features including visual map development (Xu et al., 2011) and spatial memory (Bailey et al., 2015; Qiu et al., 2010) (see also Supporting Information Tables S4 and S5) could be vital for conserving native livestock and their role in extensive agriculture, where native breeds may be subject to in situ natural selection while contributing to grazing and biodiversity maintenance priorities (Bailey et al., 2015; Halada et al., 2011; Timmermann et al., 2014).

Our results suggest native breeds may carry genetic variants important for adaptation to a rapidly changing environment due to climate change and other anthropogenic activities, and such variants could be critical for their preservation and contribute to increased environmental tolerance in commercial breeds via modern genomic techniques (O'Neill, Swain, & Kadarmideen, 2010; Rauw & Gomez‐Raya, 2015). Such advances could affect livestock survival and development related to important environmental stressors including infectious disease and parasite resistance (Kadarmideen, Ali, Thomson, Müller, & Zinsstag, 2011; O'Neill et al., 2010; Porto‐Neto et al., 2014). Our findings may also indicate trade‐offs between production gains and animal health (O'Neill et al., 2010; Rauw & Gomez‐Raya, 2015; Takasuga et al., 2015). For example, genes related to metabolism and ketosis suggest potential conflicts between human‐induced artificial selection towards high milk production and the optimal energy balance of individual animals (Mulligan & Doherty, 2008). Moreover, natural selection for more robust individuals where a higher proportion of the food intake is allocated towards energy reserve maintenance and survival (and consequently lowers productive output) merits further attention for animal welfare and long‐term evolutionary potential.

For ROH shared among six or more individuals per breed, there was limited overlap with focal genes flanking outlier SNP loci. Results supported by multiple analytical approaches will typically have higher support, although different methods with various underlying approaches can also provide diverse results (François, Martins, Caye, & Schoville, 2016; Narum & Hess, 2011; Stucki et al., 2017). The influences of selection, drift and inbreeding on ROH can be difficult to resolve (Purfield et al., 2012), in particular for small populations such as Jutland cattle. We cannot, based on our investigation of outliers and ROH, show evidence for selection of any particular trait in Jutland cattle or other native breeds. Yet taken together, the broad range of genes associated with traits under natural or human‐induced selection indicate that Jutland cattle and other native breeds represent genomic resources relevant for farming practices concerned with sustainability, animal welfare and adaptation to climate change.

There are limiting factors that should be considered for interpretation of our results, including potential ascertainment bias in the bovine array developed with a focus on commercial breeds (Supporting Information Appendix S1). Moreover, we cannot exclude the possibility that small sample sizes may have affected our results, including the analyses of outlier loci. Reports from earlier simulations with *pcadapt* indicated benefits from increasing sample sizes from 20 to 60 individuals (Luu et al., 2017). However, as certain Jutland cattle lineages now have few remaining individuals (e.g., we obtained only 16 from Westergaard and 20 from Vesterbølle), we focused on tests with equalized sample sizes and a high number of LD‐pruned loci. Accordingly, for these small populations we are confident of having included most of the genomic variation (included on the SNP array) that is still in existence.

### 4.3 The potential role of cryopreservation in preserving native breeds

A possibility emerging from our findings, consistent with recommendations from earlier studies (Charlton et al., 2018), is the potential to infuse variability into genetically depauperate lineages by means of original genetic diversity from older cryopreserved samples. These older semen samples could represent some of the existing variability prior to dramatic declines in census size and may be considered for inclusion in conservation breeding plans. For the Jutland cattle, this would appear especially relevant for the Westergaard and Vesterbølle‐lineages, where it is difficult to see management of genetically closed populations as a long‐term viable option.

## 5 CONCLUSION

Our analyses demonstrate the potential use of genomics for investigating the genetic structure and unique variation of native domestic breeds, and how these may differ from commercial breeds in traits relevant for production and environmental tolerance. Our results, using northern European extensive native cattle as a case study, also underline how these differences may have implications for human‐induced artificial and natural selection of both native and commercial breeds. We show the potential for addressing basic evolutionary inquiries and applied conservation questions that can help managers create conservation plans to preserve genetic uniqueness while maintaining in situ selection in native domestic breeds, so these can continue to be shaped by their local environmental conditions even if the *N*_E_ is low (and thus, selection has limited ability to act on the frequency of SNPs under potential selection). Furthermore, the implementation of an appropriate breeding plan will help increase the *N*_E_, which will in turn reduce the fluctuations in allelic frequencies across generations. At the same time, assortative mating strategies may be chosen for individuals that carry genes of interest, to augment the relevant allelic frequencies and reduce the risk of their loss from the population.

### CONFLICT OF INTEREST

None declared.

## DATA AVAILABILITY

Data for this study are available at https://doi.org/10.5061/dryad.hr5d4g8. Data include the PLINK files (bed, bim, fam) for 195 individual cattle and 677,311 autosomal single nucleotide polymorphism loci based on the UMD 3.1 bovine genome assembly ((https://www.ncbi.nlm.nih.gov/genome?term=bos%20taurus)) and filtered in PLINK for genotyping success of minimum 98%. Additionally, we include an Excel file with information on each of the 195 individuals, including PLINK family ID (FID) and individual ID (IID), breed/lineage and, if relevant, identity codes such as Danish studbook number.
