# Supplementary material for: Genomic analyses suggest adaptive differentiation of northern European native cattle breeds
Source: Evol Appl. 2019 Mar 12;12(6):1096–113. doi: 10.1111/eva.12783 (PMC6597895; doi:10.1111/eva.12783)
Supplement: Supplementary file 1 [file EVA-12-1096-s001.docx]

**Supplemental Materials for “Genomic analyses suggest adaptive differentiation of Northern European native cattle breeds”**

**Appendix S1**. Genetic diversity and structure in Danish Jutland cattle lineages.

Although our results indicated a distinct status for each of the four contemporary Jutland lineages, the support for differentiation was less obvious for Vesterbølle and Westergaard-lineages, and our results differed in some respects from those obtained with microsatellite markers (Brüniche-Olsen et al. 2012). We found that the Kortegaard lineage was the first Jutland cattle line that formed a unique cluster (at K = 4) and the group remained distinct, though at K = 9 it also comprised individuals from the old bulls pre-1980. Whereas our PCA results suggested an overlap between Vesterbølle and Westergaard-lineages, Oregaard and Kortegaard-lineages were highly divergent and represented the extreme positions on PC1. In contrast, microsatellite results supported Westergaard, Vesterbølle and Oregard-lineages as distinct, with Kortegaard as admixed (Brüniche-Olsen et al. 2012). The number of samples differed between the two studies, and we cannot exclude the possibility that this factor, and the inclusion or exclusion of particular individuals, may have influenced the results. However, preliminary analyses with over 100 individuals each of Kortegaard and Oregaard cattle showed similar results. Thus we do not believe incomplete sampling of these two lines to have been a limiting factor. Only few samples were available from the Vesterbølle and Westergaard-lineages, with n = 28 and n = 22 in the microsatellite study and n = 20 and n = 16 in ours, respectively. Although our sample size was slightly smaller, this is unlikely to have affected the results, considering that smaller samples of n = 9 in other breeds emerged as distinct.

Our results suggested similarities between the genomic profiles of SDM-1965 and Jutland cattle, which seem consistent with breed history (Kantanen et al., 2000; Brüniche-Olsen et al., 2012). ADMIXTURE assumes unrelated individuals, which was not feasible for our study, and the results should be interpreted with this limitation in mind. The PCA approach that does not include this assumption is thus important for comparison of population structure and within-population distribution of individual profiles (Jombart et al., 2009). Although marker type may have influenced our results, SNP profiles appear to offer substantially higher resolution than that offered by a smaller number of microsatellite loci (Kantanen et al., 2000; Brüniche-Olsen et al., 2012) toward capturing the extensive genetic drift likely to have occurred among Jutland cattle lineages. For interpretation of these results in a conservation management context, it is important to consider whether genetic divergence may be explained by genuinely different genetic variants among groups, or whether patterns emerge because some groups are more genetically depauperate overall and carry fewer genetic variants. These issues require further attention for small populations such as the Westergaard-lineage, and may help explain differentiation from other groups despite low variability.

Additionally, there are limitations to our study that should be considered for interpretation of the results. Importantly, the bovine HD chip was developed with focus on commercial breeds and traits important for dairy and meat production yield (https://www.illumina.com/Documents/products/datasheets/datasheet_bovineHD.pdf), and genetic variation represented on the array is thus expected to be biased toward these breeds (see also Stucki et al. 2017). Consequently, our assessments of genetic diversity and uniqueness in native breeds may be underestimated due to ascertainment bias. Moreover, we focused on a short 3000 bp region flanking the outlier SNPs to identify genes under potential selection, which may be a conservative assessment of genes with known or potential functional importance.

It can at times be challenging to differentiate between drift and selection (Coop et al., 2009), and despite overall encouraging results for simulation tests with *pcadapt* (Luu et al., 2016) we cannot exclude the possibility of false positives or negatives (Weigand & Leese, 2018). Yet multiple results supporting overall selective differences between breeds, such as those between commercial breeds and Jutland cattle, and between native breeds, provide support for adaptive differentiation, and we believe these findings merit further study. In contrast, only 18 of 62 outliers between the Jutland cattle Kortegaard and Oregaard lineages, which are believed to have diverged due to founder effect and genetic drift, had flanking regions exhibiting functional genes. Only few of these seemed relevant to livestock (e.g., some were linked to diseases such as cancer), and future investigation could help resolve the relevance of these findings, specifically the gene *IGF1*. However, given lack of independent (i.e, non-genetic) information on emergence of any selective differences since establishment of Kortegaard and Oregaard lineages in the 1980s, our findings should be interpreted with caution. Kortegaard and Oregaard lineages appeared quite similar from microsatellite results (Brüniche-Olsen et al., 2012), although our SNP profiles indicated strong genetic drift, which was also evident in our examination of a larger sample from each of these two lineages (Supplemental Figure S1).

Concerning sample sizes, the The Faroe Island sample is small with n=8 and it thus seems relevant and important to include equally small samples from other breeds to assess whether lack of any distinct cluster for this group is a sample size effect. Conversely, if other breeds such as Holstein and Jersey can be detected as distinct clusters with a similar sample size of n=9 based on the data being analysed, the lack of a distinct Faroe Island cluster seems unlikely to be an effect of sample size alone. Inclusion of additional individuals sampled from environments outside the North Atlantic/Nordic region (e.g., samples from global Holstein or Jersey populations) may introduce animals that have experienced differential selective pressures such as temperature (e.g. Pitt et al., 2018). Although the probability of such events may be small for commercial cattle that likely have similar conditions with high levels of human care in relatively controlled environments, the inclusion of animals only from a smaller geographic region in Northern Europe removes this possible source of uncertainty from our study. Furthermore, earlier simulations with high-density genomic profiles comprising thousands of markers and variable numbers of samples from highly structured populations have suggested that accurate estimates of genetic differentiation is well-represented with inclusion of less than 10 samples (Willing et al., 2012; Nazareno et al., 2017; Gaughran et al., 2018; Pitt et al., 2018).

An important question for future conservation breeding plans is whether it is desirable to maintain this evolutionary trajectory over the long term, as an increase of the N_E_ will clearly alleviate the risk for accumulation of mildly deleterious alleles (Demontis et al., 2009). Although our calculations of N_E_ is based on 88K loci pruned for linkage disequilibrium, assumptions including selectively neutral loci, closed populations and discrete generations (Waples and Do, 2008) may have influenced our results and should be considered when interpreting the findings.

**References**

Brüniche-Olsen, A., Gravlund, P., & Lorenzen, E.D. (2012). Impacts of genetic drift and restricted gene flow in indigenous cattle breeds: Evidence from the Jutland breed. Animal Genetic Resources, 50, 75–85.

Coop, G., Pickrell, J.K., Novembre, J., Kudaravalli, S., Absher, D., Myers, R.M., …Pritchard, J.K. (2009). The role of geography in human adaptation. *PLoS Genetics*, 5(6), e1000500.

Demontis, D., Pertoldi, C., Loeschcke, V., Mikkelsen, K., Axelsson, T., & Kristensen, T.N. (2009). Efficiency of selection, as measured by single nucleotide polymorphism variation, is dependent on inbreeding rate in *Drosophila melanogaster*. *Molecular Ecology*, 18, 4551-4563.

Gaughran, S.J., Quinzin, M.C, Miller, J.M., Garrick, R.C., Edwards, D.L., Russello, … Caccone, A. (2018). Theory, practice, and conservation in the age of genomics: The Galápagos giant tortoise as a case study. *Evolutionary Application*, 11, 1084-1093.

Jombart, T., Pontier, D., & Dufour, A.-B. (2009). Genetic markers in the playground of multivariate analysis. *Heredity*, 102, 330-341.

Kantanen, J., Olsaker, I., Holm, L.E., Lien, S., Vilkki, J., Brusgaard, K., Eythorsdottir, E., Danell, B., & Adalsteinsson, S. (2000). Genetic diversity and population structure of 20 North European cattle breeds. *The Journal of Heredity*, 91, 446–57.

Luu, K., Bazin, E., & Blum, M.G.B. (2017). pcadapt: an R package to perform genome scans for selection based on principal component analysis. Molecular Ecology Resources, 17, 67-77.

Nazareno, A.G., Bemmels, J.B.,, Dick, C.W., & Lohmann, L.G. (2017). Minimum sample sizes for population genomics: an empirical study from an Amazonian plant species. *Molecular Ecology Resources*, 17, 1136-1147.

Pitt, D., Bruford, M.W., Barbato, M., Orozco-terWengel, P., Martínez, R., & Sevane, N. (2018). Demography and rapid local adaptation shape Creole cattle genome diversity in the tropics. *Evolutionary Applications*, DOI: 10.1111/eva.12641, published 29 April 2018).

Stucki, S., Orozco-terWengel, P., Forester, B. R., Duruz, S., Colli, L., Masembe, C., … Joost, S. (2017). High performance computation of landscape genomic models including local indicators of spatial association. *Molecular Ecology Resources*, 17, 1072–1089.

Waples, R.S., & Do, C. (2008) LDNE: a program for estimating effective population size from data on linkage disequilibrium. Molecular Ecology Resources, 8, 753–756.

Weigand, H., & Leese, F. (2018). Detecting signatures of positive selection in non-model species using genomic data. *Zoological Journal of the Linnean Society*, published online 14 April 2018, doi: https://doi.org/10.1093/zoolinnean/zly007.

Willing, E.-M., Dreyer, C., & van Oosterhout, C. (2012). Estimates of genetic differentiation measured by F_ST_ do not necessarily require large sample sizes when using many SNP markers. *PLoS ONE*, 7(8), e42649.

**Appendix S2**. Outlier *loci* associated with human-induced and natural selection.

Below we summarize the findings for focal genes associated with artificial and/or natural selection, and the test(s) where they were identified. Several of the genes described below are known under multiple gene symbols. The gene symbol we identified in the search for functional bovine genes may be different from that listed in research reports on findings for other species such as mice and humans, but genes thought to represent likely homologs across taxa and associated gene symbol synonyms can be found by searching the National Center for Biotechnology Information (NCBI) web site HomoloGene (https://www.ncbi.nlm.nih.gov/homologene/). We broadly classified flanking genes considered to be under artificial selection as those known or believed to influence production traits (meat, milk), physical appearance, and reproduction. We generally considered flanking genes associated with climate adaptation, behaviour and cognition, hormones, infection and immunity, metabolism and olfaction as being under natural selection. However, both artificial and natural selection may have been influential across categories over space and time.

**Tables**

**Supplemental Table S1**. Danish stud book data for cryopreserved semen samples from pre-1980 and post-1980 individuals included for temporal comparison of genetic diversity.

| **Stud book number** | **Name** | **Birth year** | **Breed** | **Category** |
| --- | --- | --- | --- | --- |
| DK-02-7272 | ÅLB Bos | 1960 | SDM-1965 | Pre-1980 |
| DK-02-7304 | KOL Gordon | 1961 | SDM-1965 | Pre-1980 |
| DK-02-7464 | SK Hal | 1961 | SDM-1965 | Pre-1980 |
| DK-02-7480 | BJ Frans | 1961 | SDM-1965 | Pre-1980 |
| DK-02-7784 | SK Jet | 1963 | SDM-1965 | Pre-1980 |
| DK-02-7807 | HHJ Frans | 1964 | SDM-1965 | Pre-1980 |
| DK-02-7975 | HJ Swea | 1964 | SDM-1965 | Pre-1980 |
| DK-02-8050 | MRS Frans | 1961 | SDM-1965 | Pre-1980 |
| DK-02-8175 | HHJ Pau | 1966 | SDM-1965 | Pre-1980 |
| DK-02-8294 | VIB Ernst | 1966 | SDM-1965 | Pre-1980 |
| DK-02-8689 | ÅLB Horn | 1967 | SDM-1965 | Pre-1980 |
| DK-02-9011 | THY Thor | 1968 | SDM-1965 | Pre-1980 |
| DK-02-9201 | SDJ Kjær | 1968 | SDM-1965 | Pre-1980 |
| DK-02-12549 | SK Keimpe | 1976 | SDM-1965 | Pre-1980 |
| Dk-02-13887 | SK Hero | 1978 | SDM-1965 | Pre-1980 |
| DK-04-87004 | Vendelbo | 1980 | Danish Jutland cattle | Post-1980 |
| DK-04-87202 | Vilfred | 1993 | Danish Jutland cattle | Post-1980 |
| DK-04-87246 | Engdal 81 #1* | 1998 | Danish Jutland cattle | Post-1980 |
| DK-04-87226 | Engdal Tyr | 1994 | Danish Jutland cattle | Post-1980 |
| DK-04-87217 | Eng DonCII | 1995 | Danish Jutland cattle | Post-1980 |
| DK-04-87246 | Engdal 81 #2* | 1995 | Danish Jutland cattle | Post-1980 |
| DK-04-87001 | Gråblis | 1986 | Danish Jutland cattle | Post-1980 |
| DK-04-87002 | Musegrå | 1987 | Danish Jutland cattle | Post-1980 |
| DK-04-87006 | Jyden | 1982 | Danish Jutland cattle | Post-1980 |
| DK-04-87007 | Donsted | 1983 | Danish Jutland cattle | Post-1980 |
| DK-04-87008 | Vesterbøll | 1990 | Danish Jutland cattle | Post-1980 |
| DK-04-87213 | Engdal Grå | 1997 | Danish Jutland cattle | Post-1980 |
| DK-04-87242 | GRU Lagen | 2001 | Danish Jutland cattle | Post-1980 |
| DK-04-87250 | GRU Ditlev | 2002 | Danish Jutland cattle | Post-1980 |
| DK-04-87254 | GRU Maribo | 2004 | Danish Jutland cattle | Post-1980 |
| DK-02-232967 | Kongs Max | 1992 | SDM-1965 | Post-1980 |
| DK-02-234200 | Kongsbjørn | 1993 | SDM-1965 | Post-1980 |
| DK-02-242991 | GRU Fransy | 1999 | SDM-1965 | Post-1980 |
| DK-02-248885 | Ø Peter | 2004 | SDM-1965 | Post-1980 |

*Two samples were included from the Jutland bull DK-04-87213 Engdal 81

**Supplemental Table S2**. Excel file with all detected SNP loci, providing chromosome number, SNP ID, SNP basepair (bp) position, start and end point of the 3000 bp flanking region on either side, and tests T1-T7 described in the Methods and Table 2. For genes observed we report genetic function (e.g., protein coding), gene/locus symbol and NCBI description (https://www.ncbi.nlm.nih.gov/gene/), reported functional traits with links/references, and the key functions we selected for identification of focal genes.

**Supplemental Table S3**. Values for Identity-By-Descent (IBD or PI_HAT) calculated for cattle breeds and lineages based on 710,471 loci. N represents the number of individuals with the number of pairwise comparisons in parentheses.

|  | Jutland Kortegaard | Jutland Oregaard | Jutland Vester-bølle | Jutland Wester-gaard | Old bulls pre-1980^1^ | Old bulls post-1980^2^ | SDM- 1965 | WN Fjord cattle^3^ | WN Red-polled cattle^4^ | Faroe Islands | Holstein | Jersey |
| --- | --- | --- | --- | --- | --- | --- | --- | --- | --- | --- | --- | --- |
| N | 20(190) | 20(190) | 20(190) | 16(120) | 15(105) | 14(91) | 20(190) | 21(210) | 19(171) | 8(28) | 9(36) | 9(36) |
| Min | 0.000 | 0.000 | 0.000 | 0.187 | 0.000 | 0.000 | 0.000 | 0.000 | 0.000 | 0.196 | 0.209 | 0.272 |
| Max | 0.642 | 0.677 | 0.620 | 0.724 | 0.591 | 0.685 | 0.592 | 0.566 | 0.609 | 0.645 | 0.341 | 0.506 |
| Sum | 56.217 | 49.642 | 41.455 | 46.076 | 22.640 | 12.185 | 30.418 | 31.638 | 36.949 | 8.210 | 8.657 | 11.776 |
| Mean | 0.296 | 0.261 | 0.218 | 0.384 | 0.216 | 0.134 | 0.160 | 0.151 | 0.216 | 0.293 | 0.240 | 0.327 |
| Std. error | 0.01 | 0.01 | 0.01 | 0.01 | 0.01 | 0.02 | 0.01 | 0.01 | 0.01 | 0.020 | 0.01 | 0.01 |
| Variance | 0.009 | 0.016 | 0.010 | 0.015 | 0.011 | 0.028 | 0.020 | 0.008 | 0.007 | 0.011 | 0.001 | 0.003 |
| Stand. dev | 0.094 | 0.127 | 0.101 | 0.123 | 0.106 | 0.167 | 0.141 | 0.088 | 0.085 | 0.106 | 0.029 | 0.053 |
| Median | 0.276 | 0.252 | 0.215 | 0.375 | 0.180 | 0.000 | 0.193 | 0.160 | 0.198 | 0.256 | 0.232 | 0.315 |
| 25 percentile | 0.249 | 0.205 | 0.189 | 0.281 | 0.164 | 0.000 | 0.000 | 0.136 | 0.173 | 0.215 | 0.218 | 0.291 |
| 75 percentile | 0.337 | 0.327 | 0.258 | 0.450 | 0.210 | 0.275 | 0.233 | 0.183 | 0.226 | 0.336 | 0.253 | 0.339 |
| Skewness | 0.417 | 0.110 | 0.009 | 0.673 | 2.099 | 0.903 | 0.364 | 0.575 | 2.568 | 1.824 | 1.526 | 1.943 |
| Kurtosis | 3.744 | 1.393 | 2.386 | -0.022 | 5.010 | -0.124 | -0.430 | 4.039 | 9.442 | 3.681 | 2.792 | 3.989 |
| Geom. mean | 0.000 | 0.000 | 0.000 | 0.365 | 0.000 | 0.000 | 0.000 | 0.000 | 0.000 | 0.279 | 0.239 | 0.324 |
| Coeff. var | 31.916 | 48.612 | 46.381 | 32.011 | 49.225 | 124.739 | 87.789 | 58.638 | 39.188 | 36.175 | 12.161 | 16.062 |

^1^SDM-1965 cryopreserved samples

^2^Jutland cattle cryopreserved samples

^3^Western Norwegian Fjord cattle

^4^Western Norwegian Red-polled cattle

**Supplemental Table S4**. Focal genes found in the 3000 bp flanking regions of outlier single nucleotide polymorphism loci in native and commercial cattle. The T1 test included all breeds/lineages, T2 examined two Jutland cattle lineages believed to have diverged by genetic drift since the 1980s, T3 compared 1960-1980 cryopreserved Danish SDM-1965 and Holstein cattle, T4 examined 1960-1980 cryopreserved Danish SDM-1965 and Western Norwegian Red-polled cattle, T5 compared Western Norwegian Fjord cattle and Western Norwegian Red-polled cattle, T6 examined post-1980 cryopreserved Jutland cattle and Holstein cattle, and T7 compared post-1980 cryopreserved Jutland cattle and Western Norwegian Red-polled cattle (see also Table 2).

| **Category** | **Focal genes** | **Tests** | **Known or proposed function** | **Reference** |
| --- | --- | --- | --- | --- |
| Production traits (growth/meat) | *ME1*  *TRPV4*  *LRP2*  *IGF1*  *CTTNBP2NL*  *MECOM*  *PTPN1*  *PRDM16*  *ADAMTSL3*  *FLT1*  *ARHGEF3*  *CEP128*  *CYP2J2*  *ERCC6L2*  *MYOM3*  *PALM2*  *SLC8A1*  *ZNFX1*  *AKT3*  *BBX*  *COL5A1*  *DCHS2*  *DIS3L2*  *DSCAM*  *F10*  *FAP*  *FBLN7*  *GLRA1*  *GUCY1A1*  *HPS5*  *MYBPC1*  *PDE1A*  *RIN3*  *RPS6KA4*  *RUNX2*  *SMURF1*  *TENM4*  *TTN*  *BMP7*  *ESRRG*  *FBN1*  *FGD3*  *FLVCR1*  *IARS*  *PHLDB2*  *ROR2* | T1  T1  T1, T3  T2  T3  T3  T3  T4  T4  T5  T6  T6  T6  T6  T6  T6  T6  T6  T7  T7  T7  T7  T7  T7  T7  T7  T7  T7  T7  T7  T7  T7  T7  T7  T7  T7  T7  T7  T6, T7  T6, T7  T6, T7  T6, T7  T6, T7  T6, T7  T6, T7  T6, T7 | Cattle meat quality  Bovine cartilage cells  Regulation of bovine obesity  Bone mineral density  Cattle growth  Human height  Zebra fish growth  Bovine white fat cell differentiation  Human bone mineral density and osteoporotic fracture  Mouse lipid metabolism  Cattle body weight and average daily weight gain  Regulation of lean body mass in humans  Cattle body measurement traits  Human body measurement traits  Cattle meat quality and reproduction  Human bone density  Human body size and adiposity  Mouse cardiac tissue  Human bone marrow failure and microcephaly  Human striated muscle development  Human height  Bovine cardiac function  Human height  Mouse skeletal muscle development  Mouse bone strength  Human joint laxity and Ehlers-Danlos syndrome  Human skeletal strength  Human adult height  Hirschsprung’s disease with associated congenital cognitive and digestive impairments in humans  Human blood coagulation  Bovine osteoarthritis  Human cardiac effects and craniofacial abnormalities  Bovine skeletal muscle disease  Mouse lung function  Human albinism  Bovine meat marbling  Bovine growth performance  Bovine cardiac muscle  Human bone disease  Human skin inflammation  Human skeletal disorder  Human bone formation  Human hereditary essential tremor  Bovine cardiac performance  Polydactyly (various taxa)  Bovine growth traits  Human bone formation  Bovine connective tissue disorders  Human connective tissue disorders  Bovine carcass weight and skeletal dysplasia  Human and mouse congenital anemia  Human growth retardation  Mammalian skeletal muscle  Human skeletal disorder | Gill et al., 2011  Mah et al., 2016  Sanchez et al., 2014  Wang et al., 2011  Zhang et al., 2014  Okada et al., 2010  Gomez-Requeni et al., 2010  Ishibashi et al., 2012  Hwang et al., 2011  Miraldi et al., 2013  Wang et al., 2012  Urano et al., 2014  Liu et al., 2012  Liu et al., 2010  Ishida et al., 2017  Mullin et al, 2008  Croteau-Chonka et al., 2011  Hanif et al., 2017  Zhang et al., 2016  Schoenauer et al., 2008  Kim et al, 2010  Beaugé et al., 2002  van der Valk et al., 2015  Wei et al., 2013  Bassett et al., 2012  Monroe et al., 2015  Han et al., 2012  Okada et al., 2010  Jannot et al., 2013  Sun et al., 2016  Milner et al., 2006  Russell et al., 2014  Pierce et al., 2001  Bachiller et al., 2013  Carmona-Rivera et al., 2011  Tong et al., 2014  Tong et al., 2015  Vallet et al., 2015  Vallet et al., 2015  Bertelsen et al., 2011  Xu et al., 2017  Zhao et al., 2004  Hor et al., 2015  Fukuda et al., 2003  Galis et al., 2001  Huang et al., 2013  Elfassihi et al., 2010  Hirano et al., 2012  Cecchi et al., 2013  Takasuga et al., 2015  Rey et al., 2008  Orenstein et al., 2017  Proszynski & Sanes, 2013  Habib et al., 2013 |
| Milk production | *BTC*  *ABCG2*  *CTNND1*  *TCF7L2*  *CUX1*  *PDE4D*  *IGF1*  *SLC2A8/GLUT1*  *ATP1A1*  *SLC5A1*  *ITGA6* | T1  T3  T3  T3  T3  T1, T5  T2  T4  T4  T7  T1, T6, T7 | Peptide growth in bovine milk  Cattle milk quality  Cattle milk fatty acids  Veterinary pharmacology in lactating dairy animals  Mouse mammary morphogenesis  Cattle milk yield  Mouse mammary gland gene expression  Bovine mammary glands  Cattle milk fat yield  Cattle milk fatty acids  Cattle milk production  Cattle milk production, possibly also mastitis resistance  Bovine milk production and maintenance of glucose homeostasis during lactation  Dairy cattle mammary development | Dunbar et al., 1999  Otero et al., 2016  Li et al., 2016  Lindner et al., 2013  Kurley et al., 2012  Jiang et al., 2005  Maitra et al., 2006  Dostaler-Touchette et al., 2009  Zhang et al., 2014  Li et al., 2016  Gross et al., 2015  Liu et al., 2012  Zhao et al., 2005  Zhao et al., 2015 |
| Reproduction | *ITGA6*  *HSD17B12*  *SOX5*  *DPH6/ATPBD4*  *LRP2*  *BIRC5*  *IGF1*  *GABRA4*  *LHX6*  *LRRC34*  *NR3C1*  *CTTNBP2NL*  *ABCG2*  *RXFP1/LGR7*  *ELMO1*  *AVEN*  *CD9*  *EBF1*  *GSTM1*  *SPEF2*  *NPHP4*  *STAU2*  *UNC5C*  *ZNF462*  *ATP6V0A2*  *CATSPERD*  *HOOK1*  *NRG1*  *NTRK2*  *PATE1*  *RANBP9*  *UBE2E3*  *WSB2*  *ZP2*  *SPATA31A3* | T1  T1  T1  T1  T1, T3  T1, T5  T2  T3  T3  T3  T3  T3  T3  T3  T4  T6  T6  T6  T6  T6  T6  T6  T6  T6  T7  T7  T7  T7  T7  T7  T7  T7  T7  T7  T6, T7 | Cattle reproduction  Human and mouse ovarian function  Cattle semen quality  Human age at menopause  Mouse reproduction  Cattle embryo development  Cattle fertility  Cattle heat interval  Mouse onset of puberty  Mammalian reproductive behaviours  Mouse ovary development  Bovine follicular maturation  Cattle conception rates  Cattle sperm maturation  Human implantation  Mouse spermatogenesis  Bovine oocyte maturation and quality  Bovine sperm-oocyte interactions  Bovine oocyte fertilization capacity  Human preterm birth  Bovine sperm quality  Pig sperm defects and infertility  Human sperm development  Bovine oocyte maturation  Bovine conception rate and embryonic development  Human age at menarche  Human sperm motility and fertility  Mouse male fertility  Mouse spermatogenesis  Bovine uterus growth and function  Bovine sperm viability  Human sperm-oocyte interactions  Mouse spermatogenesis and fertility  Mouse testis development  Mouse gonad development and spermatogenesis  Bovine sperm-egg interactions  Bovine monospermic fertilization  Human and rat spermatogenesis | Pate et al., 2007  Kemiläinen et al., 2016  Hering & Kaminski, 2016  Pyun et al. 2014  Oh et al., 2013  Park et al., 2007  Moran et al., 2016  Hax et al., 2017  Sabaliauskas et al., 2012  Choi et al., 2005  Chen et al., 2012  Tetsuka et al., 2010  Sugimoto et al., 2013  Caballero et al., 2012  Campitiello et al., 2016  Elliott & Ravichandran, 2010  O’Shea et al., 2013  Zhou et al., 2009  Zhou et al., 2013  Zhang et al., 2017  Hering et al., 2015  Sironen et al., 2006  Won et al., 2011  Calder et al., 2008  Sugimoto et al., 2015  Perry et al., 2009  Ota et al., 2013  Chung et al., 2011  Mendoza-Lujambio et al., 2002  Akbalik & Ketani, 2013  Li et al., 2012  Margalit et al., 2012  Bao et al., 2014  Bedard et al., 2005  Sarraj et al., 2007  Ikeda et al., 2002  Burkart et al., 2012  Luk et al., 2006 |
| Physical appearance | *KRT31*  *KRTAP* | T4, T5  T7 | Keratin production (component of hair, nails, and horns)  Human hair  Evolution of hair across mammalian species | Kaytes et al., 1991; Winter et al., 1997  Rogers et al., 2004; Shibuya et al., 2004  Wu et al. 2008 |
| Climate adaptation | *NUDCD3*  *HSPBP1*  *SLC18A1/VMAT-1*  *ATP1A1*  *RPTOR*  *ACBD6* | T1  T3  T3, T4  T4  T4  T7 | Climate tolerance in *Drosophila melanogaster*  Thermal tolerance in Arabidopsis (*Arabidopsis thaliana*)  Rat hypothermia resistance  Cattle heat tolerance  Human thermogenesis and immune response  Arabidopsis cold acclimation in the related *ACBP1*  Arabidopsis drought tolerance in the related *ACBP2* | Schmidt et al., 2008  Zhang et al., 2010  Talaei et al., 2011  Liu et al., 2010; 2011  Sun et al., 2010  Du et al. 2010  Du et al. 2013 |
| Behaviour and cognition | *GRM7*  *CLSTN2*  *LSAMP*  *CERS6*  *GRID2/LOC536367*  *CHRNB2*  *DAB1*  *DSCAM*  *NETO1*  *TMEM132D* | T1  T1  T1, T5  T3  T3  T4  T7  T7  T7  T7 | Human brain function and anxiety  Mouse brain function and anxiety  Human memory and cognitive flexibility  Mouse spatial memory  Mouse ability to adapt to novel stressful environments  Mouse behavioural abnormalities  Mouse behaviour flexibility  Mouse circadian timing network  Mouse visual map development  Mouse exploration of novel environments  Mouse grooming behaviour  Mouse spatial learning  Human stress response  Mouse spatial learning and memory  Human and mouse panic disorder and anxiety | Park et al., 2013  O’Connor et al., 2013  Preuschhof et al., 2010  Qiu et al., 2010  Innos et al., 2012  Ebel et al., 2013  Dickson et al. 2010  Mendoza et al., 2010  Xu et al., 2011  Bourgeois et al., 2012  Strazielle et al., 2012  Jacquelin et al., 2012  Logue et al., 2015  Ng et al., 2009  Erhardt et al., 2011 |
| Hormones | *SLC26A4*  *SAFB2* | T1  T3 | Human thyroid disease  Human estrogen receptor | Kallel et al., 2013  Townson et al, 2003; Jiang et al., 2006 |
| Infection and immunity | *DMKN*  *TRAPPC9*  *NFATC2IP*  *SPAG11B*  *PLCXD3*  *SIGIRR*  *GZMB*  *STXBP6*  *PRODH2*  *CPNE3*  *(Ig)M/CADM2* | T1  T1, T3, T4  T3  T4  T6  T6  T7  T7  T7  T6, T7  T6, T7 | Inflammatory diseases, skin wound healing  Bovine viral diarrhea virus  Human immunity  Mouse parasite resistance  Cattle immune and reproductive functions  Association found to Creutzfeldt-Jakob disease  No association found to Creutzfeldt-Jakob disease  Mouse resistance to bacterial pathogens  Human rheumatoid arthritis  Human rheumatoid arthritis  Arabidopsis disease resistance  Bovine mastitis  Bovine immune system and immunoglobulin antibody diversification | Hasegawa et al., 2013  Zahoor et al., 2010  Benita et al., 2010  Fathman et al., 2010  Avellar et al. 2007  Bishop et al., 2013  Balendra et al., 2016  Sham et al., 2013  Darrah et al., 2017  Krintel et al., 2012  Cecchini et al., 2011  Moyes et al., 2010  Saini & Kaushik, 2002 |
| Metabolism | *RORA*  *BTC*  *GAPDHS*  *LIPF/HGL*  *PANK1*  *OXCT1*  *EBF1*  *KLHL32*  *ABCA1*  *KSR2*  *LRRC8C*  *NEGR1*  *RGS5*  *SDCCAG8*  *HACE1* | T1  T1  T1  T3  T3  T5  T6  T6  T7  T7  T7  T7  T7  T7  T6, T7 | Mouse skeletal muscle metabolism  Mouse hyperglycemia  Rat hyperglycemia  Mouse metabolism and sperm function  Human fat digestion  Mouse fasting periods  Human catabolism and ketosis, the burning of fat for energy owing to a lack of carbohydrates  Human body fat  Human body mass index  Bovine sterol homeostasis  Human and mouse regulation of energy intake and expenditure; early-onset obesity and diabetes  Human and mouse obesity, insulin resistance and adipocyte differentiation  Mouse obesity and food intake  Human obesity and food intake  Bovine rumen vascularity and feed efficiency  Human obesity  Human susceptibility to celiac disease | Lau et al. 2011  Yamamoto et al., 2008  Paz et al., 2011  Huang et al., 2017  Miled et al., 2003  Leonardi et al., 2010  Fukao et al., 2004; Shafqat et al., 2013  Chu et al., 2017  Monda et al., 2013  Farke et al., 2006  Pearce et al., 2013  Tominaga et al., 2004; Hayashi et al., 2011  Lee et al., 2012  Gamero-Villarroel et al., 2015; Antúnez-Ortiz et al., 2017  Kern et al., 2016  Sherag et al., 2010  Einarsdottir et al., 2011 |
| Sensory – olfaction | *LOC783998/OR*  *NDUFA10*  *OR11A1* | T1  T4, T5  T7 | Canine olfactory receptors  Human sensitivity to food-related odors  Human olfaction | Benbernou et al., 2011  McRae et al. 2013  Malnic et al., 2004 |
| Sensory – vison | *NPHP4*  *TENM3*  *CTNND2* | T6  T7  T6, T7 | Senior-Løken syndrome, a rare human condition causing kidney disease and a congenital eye disorder  Mammalian eye function and vision  Human myopia susceptibility | Roepman et al., 2005  Leamey et al., 2007  Lu et al., 2011 |
| Sensory – hearing | *PCDH15*  *TSPEAR* | T7  T7 | Human hearing loss  Human deafness | Xu et al., 2017  Delmaghani et al., 2012 |

**References**

Akbelik, M.E., & Ketani, M.A. (2013). Expression of epidermal growth factor receptors and epidermal growth factor, amphiregulin and neuregulin in bovine uteroplacental tissues during gestation. *Placenta*, 34, 1232-1242.

Antúnez-Ortiz, D.L., Flores-Alfaro, E., Burguete-García, A.I., Bonnefond, A., Peralta-Romero, J., Froguel, P., Espinoza-Rojo, M., & Cruz, M. (2017). Copy number variations in candidate genes and intergenic regions affect body mass index and abdominal obesity in Mexican children. *BioMed Research International*, 2017, 2432957.

Avellar, M.C.W., Honda, L., Hamil, K.G., Radhakrishnan, Y., Yenugu, S., Grossman, G., Peter Petrusz, P., French, F.S., & Hall, S.H. (2007). Novel aspects of the sperm-associated antigen 11 (*SPAG11*) gene organization and expression in cattle (*Bos taurus*). *Biology and Reproduction*, 76, 1

Bachiller, P.R., Cornog, K.H., Kato, R., Buys, E.S., & Roberts, Jr., J.D. (2013). Soluble guanylate cyclase modulates alveolarization in the newborn lung. *American Journal of Physiology-Lung Cellular and Molecular Physiology*, 305, L569-L581.

Balendra, R., Uphill, J., Collinson, C., Druyeh, R., Adamson, G., Hummerich, H., Zerr, I., Gambetti, P., Collinge, J., & Mead, S. (2016). Variants of PLCXD3 are not associated with variant or sporadic Creutzfeldt-Jakob disease in a large international study. *BMC Medical Genetics*, 17, 28.

Bao, J., Tang, C., Li, J., Zhang, Y., Bhetwal, B.P., Zheng, H., & Yan, W. (2014). RAN-binding protein 9 is involved in alternative splicing and is critical for male germ cell development and male fertility. *PLoS Genetics*, 10(12), e1004825.

Bassett, J.H.D., Gogakos, A., White, J.K., Evans, H., Jacques, R.M., van der Spek, A.H., … Williams, G.R. (2012). Rapid-throughput skeletal phenotyping of 100 knockout mice identifies 9 new genes that determine bone strength. *PLoS Genetics*, 8(8), e1002858.

Beaugé, L., Asteggiano, C., & Berberían, G. (2002). Regulation of phosphatidylinositol-4,5-biphosphate bound to the bovine cardiac Na^+^/Ca^2+^ exchanger. *Annals of the New York Academy of Sciences*, 976, 288-299.

Bedard, N., Hingamp, P., Pang, Z., Karaplis, A., Morales, C., Trasler, J., …Wing, S.S. (2005). Mice lacking the UBC4-testis gene have a delay in postnatal testis development but normal spermatogenesis and fertility. *Molecular and Cellular Biology*, 25, 6346-6354.

Benbernou, N., Robin, S., Tacher, S., Rimbault, M., Rakotomanga, M, & Galibert, F. (2011). cAMP and IP3 signaling pathways in HEK293 cells transfected with canine olfactory receptor genes. *Journal of Heredity*, 102, S47–S61.

Benita, Y., Zhifang Cao, Z., Giallourakis, C., Li, C., Gardet, A., & Xavier, R.J. (2010). Gene enrichment profiles reveal T-cell development, differentiation, and lineage-specific transcription factors including ZBTB25 as a novel NF-AT repressor. *Blood*, 115, 5376-5384.

Bertelsen, T., Iversen, L., Riis, J.L., Arthur, J.S.C., Bibby, B.M., Kragballe, K. & Johansen, C. (2011). The role of mitogen- and stress-activated protein kinase 1 and 2 in chronic skin inflammation in mice. *Experimental Dermatology*, 20, 140-145.

Bishop, M.T, Sanchez-Juan, P., & Knight, R.S.G. (2013). Splice site SNPs of phospholipase PLCXD3 are significantly associated with variant and sporadic Creutzfeldt-Jakob disease. *BMC Medical Genetics*, 14, 91.

Bourgeois, J.-P., Meas-Yeadid, V, Lesourd, A.M., Faure, P., Pons, S., Maskos, U., … Granon, S. (2012). Modulation of the mouse prefrontal cortex activation by neuronal nicotinic receptors during novelty exploration but not by exploration of a familiar environment. *Cerebral Cortex*, 22,1007-1015.

Brüniche-Olsen, A., Gravlund, P., & Lorenzen, E.D. (2012). Impacts of genetic drift and restricted gene flow in indigenous cattle breeds: Evidence from the Jutland breed. *Animal Genetic Resources*, 50, 75–85.

Burkart, A.D., Xiong, B., Baibakov, B., Jiménez-Movilla, M., & Dean, J. (2012). Ovastacin, a cortical granule protease, cleaves ZP2 in the zona pellucida to prevent polyspermy. *Journal of Cell Biology*, 197, 37-44.

Caballero, J., Frenette, G., D’Amours, O., Dufour, M., Oko, R., & Sullivan, R. (2012). ATP-binding cassette transporter G2 activity in the bovine spermatozoa is modulated along the epididymal duct and at ejaculation. *Biology of Reproduction*, 86(6), 1-11.

Calder, M.D., Madan, P., & Watsen, A.J. (2008). Bovine oocytes and early embryos express Staufen and ELAVL RNA-binding proteins. *Zygote*, 16, 161-168.

Campitiello, M.R., Caprio, F., Mele, D., D’eufemia, D., Colacurci, N., & De Franciscis, P. (2016). Endometrial LGR7 expression and implantation failure. *Gynecological Endocrinology*, 32, 449-452.

Carmona-Rivera, C., Golas, G., Hess, R., Cardillo, N.D., Martin, E., O’Brien, K., … Gahl, W.A. (2011). Clinical, molecular and cellular features of non-Puerto Rican Hermansky-Pudlak syndrome patients of Hispanic descent. *Journal of Investigative Dermatology*, 131, 2394-2400.

Cecchi, A., Ogawa, N., Martinez, H.R., Carlson, A., Fan, Y., Penny, D.J., … Milewicz, D.M. (2013). Missense mutations in *FBN1* exons 41 and 42 cause Weill-Marchesani syndrome with thoracic aortic disease and Marfan syndrome. *American Journal of Medical Genetics Part A*, 161A, 2305-2310.

Cecchini, N.M., Monteoliva, M.I., & Alvarez, M.E. (2011). Proline dehydrogenase contributes to pathogen defense in Arabidopsis. *Plant Physiology*, 155, 1947-1959.

Chen, H., Palmer, J.S., Thiagarajan, R.D., Dinger, M.E., Lesieur, E., Chiu, H., …Wilhelm, D. (2012). Identification of novel markers of mouse fetal ovary development. PLoS ONE 7(7): e41683.

Choi, G.B., Dong, H., Murphy, A.J., Valenzuela, D.M., Yancopoulos, G.D., Swanson, L.W., & Anderson, D.J. (2005). Lhx6 delineates a pathway mediating innate reproductive behaviors from the amygdala to the hypothalamus. *Neuron*, 46, 647-660.

Chu, A.Y., Deng, X., Fisher, V.A., Drong, A., Zhang, Y., Feitosa, M.F., ….Fox, C.S. (2017). Multiethnic genome-wide meta-analysis of ectopic fat depots identifies loci associated with adipocyte development and differentiation. *Nature Genetics*, 49, 125-130.

Chung, J.-J., Navarro, B., Krapivinsky, G., Krapivinsky, L., & Clapham, D.E. (2011). A novel gene required for male fertility and functional CATSPER channel formation in spermatozoa. *Nature Communications*, 11, 153.

Croteau-Chonka, D.C., Marvelle, A.F., Lange, E.M., Lee, N.R., Adair, L.S., Lange, L.A., & Mohlke, K.E. (2011). Genome-wide association study of anthropometric traits and evidence of interactions with age and study year in Filipino women. *Obesity*, 19, 1019-1027.

Darrah, E., Kim, A., Zhang, X., Boronina, T, Cole, R.N., Fava, … & Rosen, A. (2017). Proteolysis by granzyme B enhances presentation of autoantigenic peptidylarginine deiminase 4 epitopes in rheumatoid arthritis. *Journal of Proteome Research*, 16, 355-365.

Das, S.B., Dinh, C., Shah, S., Olson, D., Ross, A., Selvakumar, P., & Sharma, R.K. (2007). Calmodulin-dependent cyclic nucleotide phosphodiesterase (PDE1) splice variants from bovine cardiac muscle. *Gene*, 396, 283-292.

Delmaghani, S., Aghaie, A., Michalski, N., Bonnet, C., Weil, D., & Petit, C. (2012). Defect in the gene encoding the EAR/EPTP domain-containing protein TSPEAR causes DFNB98 profound deafness. *Human Molecular Genetics*, 21, 3835-3844.

Dickson, P.E., Rogers, T.D., Del Mar, N., Martin, L.A., Heck, D., Blaha, C.D., Goldowitz, D., & Mittleman, G. (2010). Behavioral flexibility in a mouse model of developmental cerebellar Purkinje cell loss. *Neurobiology of Learning and Memory*, 94, 220–228.

Dostaler-Touchette, V., Bédard, F., Guillemette, C., Pothier, F., Chouinard, P.Y., & Richard, F.J. (2009). Cyclic adenosine monophosphate (cAMP)-specific phosphodiesterase is functional in bovine mammary gland. *Journal of Dairy Science*, 92, 3757-3765.

Du, Z.-Y., Chen, M.-X., Chen, Q.-F., Xiao, S., & Chye, M.-L. (2013). Overexpression of Arabidopsis acyl-CoA-binding protein ACBP2 enhances drought tolerance. *Plant, Cell and Environment*, 36, 300-314.

Du, Z.-Y., Xiao, S., Chen, Q.-F., & Chye, M.-L. (2010). Depletion of the membrane-associated Acyl-Coenzyme A-binding protein ACBP1 enhances the ability of cold acclimation in Arabidopsis^1[OA]^. *Plant Physiology*, 152, 1585-1597.

Dunbar, A.J., Priebe, I.K., Belford, D.A., & Goddard, C. (1999). Identification of betacellulin as a major peptide growth factor in milk: purification, characterization and molecular cloning of bovine betacellulin. *Biochemical Journal*, 344, 713-721.

Ebel, P., vom Dorp, K., Petrasch-Parwez, E., Zlomuzica, A., Kinugawa, K., Mariani, J., … Willecke, K. (2013). Inactivation of ceramide synthase 6 in mice results in an altered sphingolipid metabolism and behavioral abnormalities. *The Journal of Biochemistry*, 288, 21433-21447.

Einarsdottir, E., Bevova, M.R., Zhernakova, A., Monsuur, A., Koskinen, L.L.E., van’t Slot, R., … Saavalainen, P. (2011). *European Journal of Human Genetics*, 19, 682-686.

Elfassihi, L., Giroux, S., Bureau, A., Laflamme, N., Cole, D.E.C., & Rousseau, F. (2010). Association with replication between estrogen-related receptor γ (ESRRγ) polymorphisms and bone phenotypes in women of European ancestry. *Journal of Bone and Mineral Research*, 25, 901-911.

Erhardt, A., Czibere, L., Roeske, D., Lucae, S., Unschuld, P.G., Ripke, S., …Binder, E.B. (2011). TMEM132D, a new candidate for anxiety phenotypes: evidence from human and mouse studies. *Molecular Psychiatry*, 16, 647-663.

Farke, C., Viturro, E., Meyer, H.H.D., & Albrecht, C. (2006). Identification of the bovine cholesterol efflux regulatory protein ABCA1 and its expression in various tissues. *Journal of Animal Science*, 84, 2887-2894.

Fathman, J.W., Gurish, M.F., Hemmers, S., Bonham, K., Friend, D.S., Grusby, M.J., Glimcher, L.H., & Mowen, K.A. (2010). NIP45 controls the magnitude of the type 2 T helper cell response. *Proceedings of the National Academy of Sciences of the United States of America*, 107, 3663-3668.

Fukao, T., Shintaku, H., Kusubae, R., Zhang, G.X., Nakamura, K., Kondo, M., & Kondo, N. (2004). Patients homozygous for the T435N mutation of Succinyl-CoA:3-Ketoacid CoA Transferase (SCOT) do not show permanent ketosis. *Pediatric Research*, 56, 858-863.

Fukuda, N., Wu, Y., Farman, G., Irving, T.C., & Granzier, H. (2003). Titin isoform variance and length dependence of activation in skinned bovine cardiac muscle. *The Journal of Physiology*, 553, 147-154.

Gamero-Villarroel, C., González, L.M., Gordillo, I., Carrillo, J.A., García-Herráiz, A., Flores, I, Rodríguez-López, R., & Gervasini, G. (2015). Impact of NEGR1 genetic variability on psychological traits of patients with eating disorders. *The Pharmacogenomics Journal*, 15, 278-283.

Gill, J.L., Bishop, S.C., McCorquodale, C., Williams, J.L., & P. Wiener, P. (2011). Identification of polymorphisms in the malic enzyme 1, NADP(+)-dependent, cytosolic and nuclear receptor subfamily 0, group B, member 2 genes and their associations with meat and carcass quality traits in commercial Angus cattle. *Animal Genetics*, 43, 88-92.

Gomez-Requeni, P., Conceicao, L.E.C., Olderbakk Jordal, A.-E., & Rønnestad, I. (2010). A reference growth curve for nutritional experiments in zebrafish (*Danio rerio*) and changes in whole body proteome during development. *Fish Physiology and Biochemistry*, 36, 1199-1215.

Gross, J.J., van Dorland, H.A., Wellnitz, O., & Bruckmaier, R.M. (2015). Glucose transport and milk secretion during manipulated plasma insulin and glucose concentrations and during LPS-induced mastitis in dairy cows. *Animal Physiology and Animal Nutrition*, 99, 747-756.

Habib, R., Amin-ud-din, M., & Ahmad, W. (2013). A nonsense mutation in the gene *ROR2* underlying autosomal dominant brachydactyly type B. *Clinical Dysmorphology*, 22,47-50.

Han, Y., Pei, Y., Liu, Y., Zhang, L., Wu, S., Tian, Q.,... Deng, H. (2012). Bivariate genome-wide association study suggests fatty acid desaturase genes and cadherin DCHS2 for variation of both compressive strength index and appendicular lean mass in males. *Bones*, 51, 1000-1007.

Hanif, A., Edin, M.L., Zeldin, D.C., Morisseau C, Falck, J.R., & Nayeem, M.A. (2017). Vascular endothelial overexpression of human CYP2J2 (Tie2-CYP2J2 Tr) modulates cardiac oxylipin profiles and enhances coronary reactive hyperemia in mice. *PLoS ONE*, 12(3), e0174137.

Hasegawa, M., Higashi, K., Matsushita, T., Hamaguchi, Y., Saito, K., Fujimoto, M., & Takehara, K. (2013). Dermokine inhibits ELR^+^CXC chemokine expression and delays early skin wound healing. *Journal of Dermatological Science*, 70, 34–41.

Hax, L.T., Schneider, A., Jacometo, C.B, Mattei, P., Casarin da Silva, T., Farina, G., & Corrêa, M.N. (2017). Association between polymorphisms in somatotropic axis genes and fertility of Holstein dairy cows. *Therogenology*, 88, 67-72.

Hayashi, T., Nozaki, Y., Nishizuka, M., Ikawa, M., Osada, S., & Imagawa, M. (2011). Factor for adipocyte differentiation 158 gene disruption prevents the body weight gain and insulin resistance induced by a high-fat diet. *Biological and Pharmaceutical Bulletin*, 34, 1257-1263.

Hering, D.M, & Kaminski, S. (2016). Association between SOX5 genotypes and semen quality in Polish Holstein-Friesian bulls. *Polish Journal of Veterinary Sciences*, 19, 651-653.

Hering, D.M., Lecewicz, M., Kordan, W., Majewska, A., & Kaminski, S. (2015). Missense mutation in glutathione-S-transferase M1 gene is associated with sperm motility and ATP content infrozen-thawed semen of Holstein-Friesian bulls. *Animal Reproduction Science*, 159, 94-97.

Hirano, T., Matsuhashi, T., Kobayashi, N., Watanabe, T., & Sugimoto, Y. (2012). Identification of an *FBN1* mutation in bovine Marfan syndrome-like disease. *Animal Genetics*, 43, 11-27.

Hor, H., Francescatto, L., Bartesaghi, L, Ortega-Cubero, S., Kousi, M., Lorenzo-Betancor, O., … Estivill, X. (2015). Missense mutations in *TENM4*, a regulator of axon guidance and central myelination, cause essential tremor. *Human Molecular Genetics*, 24, 5677-5686.

Huang, Z., Danshina, P.V., Mohr, K., Qu, W., Goodson, S.G., O’Connell, T.M., & O’Brien, D.A. (2017). Sperm function, protein phosphorylation, and metabolism differ in mice lacking successive sperm-specific glycolytic enzymes. *Biology of Reproduction*, 97, 586–597.

Huang, Y.-Z., Wang, X.-L., He, H., Lan, X.-Y., Lei, C.-Z., Zhang, C.-L., & Chen, H. (2013). Identification and genetic effect of haplotype in the bovine *BMP7* gene. *Gene*, 532, 281-287.

Hwang, J.Y., Lee, S.H., Go, M.J., Kim, B.-J., Kou, I., Ikegawa, S., …Koh, J.-M. (2013). Meta-analysis identifies a *MECOM* gene as a novel predisposing factor of osteoporotic fracture. *Journal of Medical Genetics*, 50, 212-219.

Ikeda, K., Yonezawa, N., Naoi, K., Katsumata, T., Hamano, S., & Nakano, M. (2002). Localization of N-linked carbohydrate chains in glycoprotein ZPA of the bovine egg zona pellucida. *European Journal of Biochemistry*, 269, 4257-4266.

Innos, J., Philips, M.-A., Rauda, S., Lilleväli, K., Kõksa, S., & Vasara, E. (2012). Deletion of the Lsamp gene lowers sensitivity to stressful environmental manipulations in mice. *Behavioural Brain Research*, 228, 74– 81.

Ishibashi, J., Firtina, Z., Rajakumari, S., Wood, K.H., Conroe, H.M., Steger, D.J., & Seale, P. (2012). An evi1 –C/EBPβ complex controls peroxisome proliferator-activated receptor γ2 gene expression to initiate white fat cell differentiation. *Molecular and Cell Biology*, 32, 2289-2299.

Ishida, T., Noda, K., Jomane, F.N., & Tokunaga, T. (2017). Polymorphisms of *RDH16* and *VEGFR1* influence *M. trapezium* steatosis in Japanese Black carcass. *Animal Science Journal*, 88, 1037-1041.

Jacquelin, C., Strazielle, C., & Lalonde, R. (2012). Spontaneous alternation and spatial learning in *Dab1^scm^* (*scrambler*) mutant mice. *Brain Research Bulletin*, 87, 383-386.

Jannot, A.-S., Pelet, A., Henrion-Caude, A., Chaoui, A., Masse-Morel, M., Arnold, S., …Lyonnet, S. (2013). Chromosome 21 scan in Down syndrome reveals DSCAM as a predisposing locus in Hirschsprung disease. *PLoS ONE*, 8(5), e62519.

Jiang, S., Meyer, R., Kang, K., Osborne, C.K., Wong, J., & Oesterreich, S. (2006). Scaffold attachment factor SAFB1 suppresses estrogen receptor α-mediated transcription in part via interaction with nuclear receptor corepressor. *Molecular Endocrinology*, 20, 311–320.

Jiang, Z., De, S., Garcia, M.D., Griffin, K.B., Wu, X.-L., Xiao, Q., …Jansen, G.B. (2005). An independent confirmation of a quantitative trait locus for milk yield and composition traits on bovine chromosome 26. *Journal of Animal Breeding and Genetics*, 122, 281-284.

Kallel, R., Niasme-Grare, M., Belguith-Maalej, S., Mnif, M., Abid, M., Ayadi, H., & Hadj Kacem, H. (2013). Screening of *SLC26A4* gene in autoimmune thyroid diseases. *International Journal of Immunogenetics*, 40, 284–291.

Kaytes, P.S., McNab, A.R., Rea, T.J., Groppi, V., Kawabe, T.T., Buhl, A.E., … Vogeli, G. (1991). Hair-expression keratins: characterization and expression of a mouse type 1 keratin gene. *The Journal for Investigative Dermatology*, 97, 835-842.

Kemiläinen, H., Adam, M., Mäki-Jouppila, J., Damdimopoulou, P., Damdimopoulos, A.E., Kere, J., … Poutanen, M. (2016). The hydroxysteroid (17β) dehydrogenase family gene HSD17B12 is involved in the prostaglandin synthesis pathway, the ovarian function, and regulation of fertility. *Endocrinology*, 157, 3719-3730.

Kern, R.J., Zarek, C.M., Lindholm-Perry, A.K., Kuehn, L.A., Snelling, W.M., Freetly, H.C., Cunningham, H.C., & Meyer, A.M. (2016). Ruminal expression of the NQO1, RGS5, and ACAT1 genes may be indicators of feed efficiency in beef steers. *Animal Genetics*, 48, 90–92.

Kim, J.-J., Lee, H.-I., Park, T., Kim, K., Lee, J.-E., Cho, N.H., ... Lee, J.-K. (2010). Identification of 15 loci influencing height in a Korean population. *Journal of Human Genetics*, 55, 27-31.

Krintel, S.B., Essioux, L., Wool, A., Johansen, J.S., Schreiber, E., Zekharya, T., Akiva, P., Østergaard, M., & Hetland, M.L. (2012). CD6 and syntaxin binding protein 6 variants and response to tumor necrosis factor alpha inhibitors in Danish patients with rheumatoid arthritis. *PLoS ONE*, 7(6), e38539.

Kurley, S.J., Bierie, B, Carnahan, R.H., Lobdell, N.A., Davis, M.A., Hofman, I., … Reynolds, A.B. (2012). p120-catenin is essential for terminal end bud function and mammary morphogenesis. *Development*, 139, 1754-1764.

Lau, P., Fitzsimmons, R.L., Pearen, M.A., Watt, M.J., & Muscat, G. E. O. (2011). Homozygous staggerer (*sg/sg*) mice display improved insulin sensitivity and enhanced glucose uptake in skeletal muscle. *Diabetologia*, 54, 1169–1180.

Leamey, C.A., Merlin, S., Lattouf, P., Sawatari, A., Zhou, X., Glendining, K.A., Oohashi, T., Sur, M., & Fässler, R. (2007). Ten_m3 regulates eye-specific patterning in the mammalian visual pathway and is required for binocular vision. *PLoS Biology*, 5(9), e241.

Lee, A.W.S., Hengstler, H., Schwald, K., Berriel-Diaz, M., Loreth, D., Kirsch, M., … Schäfer, M.K.E. (2012). Functional inactivation of the genome-wide association study obesity gene neuronal growth regulator 1 in mice causes a body mass phenotype. *PLoS ONE*, 7(7), e41537.

Leonardi, R., Rehg, J.E., Rock, C.O., & Jackowski, S. (2010). Pantothenate kinase 1 is required to support the metabolic transition from the fed to the fasted state. *PLoS ONE*, 5(6), e11107.

Li, C., Li, C., Zhu, X., Wang, C., Liu, Z., Li, W., Lu, C, & Zhou, X. (2012). The expression and putative role of brain-derived neurotrophic factor and its receptor in bovine sperm. *Theriogenology*, 77, 636-643.

Li, C., Sun, D., Zhang, S., Yang, S., Alim, M.A., Zhang, Q., Li, Y., & Liu, L. (2016). Genetic effects of *FASN, PPARGC1A, ABCG2* and *IGF1* revealing the association with milk fatty acids in a Chinese Holstein cattle population based on a post genome-wide association study. *BMC Genetics*, 17:110.

Lindner, S, Halwachs, S, Wassermann, L., & Honscha, W. (2013). Expression and subcellular localization of efflux transporter ABCG2/BCRP in important tissue barriers of lactating dairy cows, sheep and goats. *The Journal of Veterinary Pharmacology and Therapeutics*, 36, 562-570.

Liu, J.Z, Medland, S.E., Wright, M.J., Heath, A.C., Madden, P.A.F., Duncan, A., …McRae, A.F. (2010). Genome-wide association of height and body mass index in Australian twin families. *Twin Research and Human Genetics*, 13, 179-193.

Liu, Y., Li, D., Li, H., Zhou, X., & Wang, G. (2011). A novel SNP of the *ATP1A1* gene is associated with heat tolerance traits in dairy cows. *Molecular Biology Reports*, 38, 83-88.

Liu, Y.X., Xu, C.H., gao, T.Y., & Sun, Y. (2012). Polymorphisms of the *ATP1A1* gene associated with mastitis I dairy cattle. *Genetics and Molecular Research*, 11, 651-660.

Liu, Y., Zan, L., Zhao, S., Xin, Y., Jiao, Y., & Li, K. (2012). Molecular characterization, expression pattern, polymorphism and association analysis of bovine *ADAMTSL3* gene. *Molecular Biology Reports*, 39, 1551-1560.

Liu, Y.X., Zhou, X., Li, D.Q., Cui, Q.W., & Wang, G.L. (2010). Association of ATP1A1 gene polymorphism with heat tolerance traits in dairy cattle. *Genetics and Molecular Research*, 9, 891-896.

Logue, M.W., Smith, A.K., Baldwin, C., Wolf, E.J., Guffanti, G., Ratanatharathorn, A., …Miller, M.W. (2015). An analysis of gene expression in PTSD implicates genes involved in the glucocorticoid receptor pathway and neural responses to stress. *Psychoneuroendocrinology*, 57, 1-13.

Lu, B., Jiang, D., Wang, P., Gao, Y., Sun, W., Xiao, X., Li, S., Jia, X., Guo, X., & Zhang, Q. (2011). Replication study supports CTNND2 as a susceptibility gene for high myopia. *Investigative Ophthalmology & Visual Science*, 52, 8258-8261.

Luk, J.M., Lee, N.P.Y., Shum, C.K., Lam, B.Y., Siu, A.F.M., Che, C.-M., …Yeung, W.S.B. (2006). Acrosome-specific gene *AEP1*: Identification, characterization and roles in spermatogenesis. *Journal of Cellular Physiology*, 209, 755-766.

Mah, W., Sonkusare, S.K., Wang, T., Azeddine, B., Pupavac, M., Carrot-Zhang, J., … Séguin, C. (2016). Gain-of-function mutation in *TRPV4* identified in patients with osteonecrosis of the femoral head. *Journal of Medical Genetics*, 53, 705-709.

Maitra, U., Seo, J., Lozano, M.M., & Dudley, J.P. (2006). Differentiation-induced cleavage of Cutl1/CDP generates a novel dominant-negative isoform that regulates mammary gene expression. *Molecular and Cellular Biology*, 26, 7466-7478.

Malnic, B., Godfrey, P.A., & Buck, L.B. (2004). The human olfactory receptor gene family. *Proceedings of the National Academy of Sciences of the United States of America*, 101, 2584-2589.

Margalit, M., Yogev, L., Yavetz, H., Lehavi, O., Hauser, R., Botchan, A.,… Kleiman, S.E. (2012). Involvement of the prostate and testis expression (PATE)-like proteins in sperm–oocyte interaction. *Human Reproduction*, 27, 1238-1248.

McRae, J.F., Jaeger, S.R, Bava, C.M., Beresford, M.K., Hunter, D. Jia, Y., … Newcomb, R.D. (2013). Identification of regions associated with variation in sensitivity to food-related odors in the human genome. *Current Biology*, 23, 1596-1600.

Mendoza, J., Pévet, P., Felder-Schmittbuhl, M.P., Bailly, Y., & Challet, E. (2010). The cerebellum harbors a circadian oscillator involved in food anticipation. *The Journal of Neuroscience*, 30, 1894 -1904.

Mendoza-Lujambio, I., Burfeind, P., Dixkens, C., Meinhardt, A., Hoyer-Fender, S., Engel, W., & Neesen, J. (2002). The *Hook1* gene is non-functional in the abnormal spermatozoon head shape (*azh*) mutant mouse. *Human Molecular Genetics*, 11, 1647-1658.

Miled, N., Bussetta, C., De caro, A., Rivière, M., Berti, L., & Canaan, S. (2003). Importance of the lid and cap domains for the catalytic activity of gastric lipases. *Comparative Biochemistry and Physiology Part B*, 136, 131–138.

Milner, J.M., Kevorkian, L., Young, D.A., Jones, D., Wait, R., Donell, S.T., …Cawston, T.E. (2006). Fibroblast activation protein alpha is expressed by chondrocytes following a pro-inflammatory stimulus and is elevated in osteoarthritis. *Arthritis Research & Therapy*, 8, R23.

Miraldi, E.R., Sharfi, H., Friedline, R.H., Johnson, H., Zhang, T., Lau, K.S., …White, F.M. (2013). Molecular network analysis of phosphotyrosine and lipid metabolism in hepatic PTP1b deletion mice. *Integrative Biology*, 5, 940-963.

Monda, K.L., Chen, G.K., Taylor, K.C., Palmer, C., Edwards, T.L., Lange, L.A., …Haiman, C.A. (2013). A Meta-Analysis Identifies New Loci Associated with Body Mass index in Individuals of African Ancestry. *Nature Genetics*, 45, 690-696.

Monroe, G.R., Harakalova, M., van der Crabben, S.N., Majoor-Krakauer, D., Bertoli-Avella, A.M., Moll, F.L., … Baas, A.F. (2015). Familial Ehlers-Danlos syndrome with lethal arterial events caused by a mutation in COL5A1. *American Journal of Medical Genetics Part A*, 167A, 1196–1203.

Moran, B., Cummins, S.B., Creevey, C.J., & Butler, S.T. (2016). Transcriptomics of liver and muscle in Holstein cows genetically divergent for fertility highlight differences in nutrient partitioning and inflammation processes. *MBC Genomics*, 17, 603.

Moyes, K.M., Drackley, J.K., Morin, D.E., & Loor, J.J. (2010). Greater expression of TLR2, TLR4, and IL6 due to negative energy balance is associated with lower expression of HLA-DRA and HLA-A in bovine blood neutrophils after intramammary mastitis challenge with Streptococcus uberis. *Functional & Integrative Genomics*,10, 53–61.

Mullin, B.H., Prince, R.L., Dick, I.M., Hart, D.J., Spector, T.D., Dudbridge, F., & Wilson, S.G. (2008). Identification of a role for the ARHGEF3 gene in postmenopausal osteoporosis. *The American Journal of Human Genetics*, 82, 1262-1269.

Ng, D., Pitcher, G.M., Szilard, R.K., Sertié, A., Kanisek, M., Clapcote, S.J., …McInnes, R.R. (2009). Neto1 is a novel CUB-domain NMDA receptor–interacting protein required for synaptic plasticity and learning. *PLoS Biology*, 7(2), e1000041.

O’Connor, R.M., Thakker, D.R., Schmutz, M., van der Putten, H., Hoyer, D., Flor, P.J., & Cryan, J.F. (2013). Adult siRNA-induced knockdown of mGlu7 receptors reduces anxiety in the mouse. *Neuropharmacology*, 72, 66-73.

Oh, S.J., Kim, T.H., Lim, J.M., & Jeong, J.-W. (2013). Progesterone induces expression of Lrp2 in the murine uterus. *Biochemical and Biophysical Research Communications*, 441, 175-179.

Okada, Y., Kamatani, Y., Takahashi, A., Matsuda, K., Hosono, N., Ohmiya, H., …Kamatani, N. (2010). A genome-wide association study in 19 633 Japanese subjects identified *LHX3-QSOX2* and *IGF1* as adult height loci. *Human Molecular Genetics*, 19, 2303-2312.

Orenstein, N., Weiss, K., Oprescu, S.N., Shapira, R., Kidron, D., Vanagaite-Basel, L., Antonellis, A., & Muenke, M. (2017). Bi-allelic *IARS* mutations in a child with intra-uterine growth retardation, neonatal cholestasis, and mild developmental delay. *Clinical Genetics*, 91, 913-917.

O’Shea, L.C., Hensey, C., & Fair, T. (2013). Progesterone regulation of AVEN protects bovine oocytes from apoptosis during meiotic maturation. *Biology of Reproduction*, 89, 146.

Ota, K., Jaiswal, M.K., Ramu, S., Jeyendran, R., Kwak-Kim, J., Gilman-Sachs, A., & Beaman, K.D. (2013). Expression of a2 vacuolar ATPase in spermatozoa is associated with semen quality and chemokine-cytokine profiles in infertile men. *PLoS ONE*, 8(7), e70470.

Otero, J.A., Miguel, V., Gonzalez-Lobato, L., Garcia-Villalba, R., Espin, J.C., Prieto, J.G., Merino, G., & Alvarez, A.I. (2016). Effect of bovine ABCG2 polymorphism Y581S SNP on secretion into milk of enterolactone, riboflavin and uric acid. *Animal*, 10, 238-247.

Park, S., Jung, S.-W., Kim, B.-N., Cho, S.-C., Shin, M.-S., Kim, J.-W., & Kim, H.-W. (2013). Association between the GRM7 rs3792452 polymorphism and attention deficit hyperacitiveity disorder in a Korean sample. *Behavioral and Brain Functions*, 9, 1.

Park, S.Y., Kim, E.Y, Jeon, K., Cui, X.-S., Lee, W.D., Kim, N.-H., Park, S.P., & Lim, J.H. (2007). Survivin acts as anti-apoptotic factor during the development of bovine pre-implantation embryos. *Molecular Reproduction and Development*, 74, 582-590.

Pate B.J., White, K.L., Winger, Q.A., Rickords, L.F., Aston, K.I., Sessons, B.R., …Bunch T.D. (2007). Specific integrin subunits in bovine oocynes, including novel sequences for alpha 6 and beta 3 subunits. *Molecular Reproduction and Development*, 74, 600-607.

Paz, A.H., Salton, G.D., Ayala-Lugo, A., Gomes, C., Terraciano, P., Scalco, R., …Cirne-Lima1, E. (2011). Betacellulin overexpression in mesenchymal stem cells induces insulin secretion in vitro and ameliorates streptozotocin-induced hyperglycemia in rats. *Stem Cells and Development*, 20, 223-232.

Pearce, L.R., Atanassova, N., Banton, M.C., Bottomley, B., van der Klaauw, A.A., Revelli, J.-P., … Farooqi, I.S. (2013). KSR2 mutations are associated with obesity, insulin resistance, and impaired cellular fuel oxidation. *Cell*, 155, 765–777.

Perry, J.R.B., Stolk, L., Franceschini, N., Lunetta, K.L., Zhai, G., McArdle, P.F., … Murabito, J.M. (2009). Meta-analysis of genome-wide association data identifies two loci influencing age at menarche. *Nature Genetics*, 41, 648-650.

Pierce, K.D., Handford, C.A., Morris, R., Vafa, B., Dennis, J.A., Healy, P.J., & Schofield, P.R. (2001). A nonsense mutation in the α1 subunit of the inhibitory glycine receptor associated with bovine myoclonus. *Molecular and Cellular Neuroscience*, 17, 354-363.

Preuschhof, C., Heekerena, H.R, Li, S.-C., Sanderc, T., Lindenberger, U., & Bäckmand, L. (2010). KIBRA and CLSTN2 polymorphisms exert interactive effects on human episodic memory. *Neuropsychologia*, 48, 402–408.

Proszynski, T.J., & Sanes, J.R. (2013). Amotl2 interacts with LL5β, localizes to podosomes and regulates postsynaptic differentiation in muscle. *Journal of Cell Science*, 126, 2225-2235.

Pyun, J.A., Kim, S., Cho, N.H., Koh, I., Lee, J.Y., Shin, C., & Kwack, K. (2014). Genome-wide association studies and epistasis analyses of candidate genes related to age at menarche and age at natural menopause in a Korean population. *Menopause*, 21, 522-529.

Qiu, S., Champagne, D.L., Peters, M., Catania, E.H., Weeber, E.J., Levitt, P., & Pimenta, A.F. (2010). Loss of limbic system-associated membrane protein leads to reduced hippocampal mineralocorticoid receptor expression, impaired synaptic plasticity and spatial memory deficit. *Biological Psychiatry*, 68, 197-204.

Rey, M.A., Duffy, S.P., Brown, J.K., Kennedy, J.A., Dick, J.E., Dror, Y., & Tailor, C.S. (2008). Enhanced alternative splicing of the *FLVCR1* gene in Diamond Blackfan anemia disrupts *FLVCR1* expression and function that are critical for erythropoiesis. *Haematologica*, 93, 1617-1626.

Roepman, R., Letteboer, S.J.F., Arts, H.H., van Beersum, S.E.C., Lu, X., Krieger, E., Ferreira, P.A., & Cremers, F.P.M. (2005). Interaction of nephrocystin-4 and RPGRIP1 is disrupted by nephronophthisis or Leber congenital amaurosis-associated mutations. *Proceedings of the National Academy of Sciences of the United States of America*, 102, 18520-18525.

Rogers, M.A., Langbein, L., Winter, H., Beckmann, I., Praetzel, S., & Schweizer, J. (2004). Hair keratin associated proteins: characterization of a second high sulfur KAP gene domain on human chromosome 21. *The Journal of Investigative Dermatology*, 122, 147-158.

Russell, M.W., Raeker, M.O., Geisler, S.B., Thomas, P.E., Simmons, T.A., Bernat, J.A., & Innis, J.W. (2014). Functional analysis of candidate genes in 2q13 deletion syndrome implicates FBLN7 and TMEM87B deficiency in congenital heart defects and FBLN7 in craniofacial malformations. *Human Molecular Genetics*, 23, 4272-4284.

Sabaliauskas, N., Shen, H., Homanics, G.E., Smith, S.S., & Aoki, C. (2012). Knockout of the γ-aminobutyric acid receptor subunit α4 reduces functional δ-containing extrasynaptic receptors in hippocampal pyramidal cells at the onset of puberty. *Brain Research*, 1450, 11-23.

Saini, S.S., & Kaushik, A. (2002). Extensive CDR3H length heterogeneity exists in bovine foetal VDJ rearrangements. *Scandinavian Journal of Immunology*, 55, 140-148.

Sanchez, J.C., Lopez-Zapata, D.F., & Wilkins, R.J. (2014). TRVP4 channels activity in bovine articular chondrocutes: Regulation by obesity-associated mediators. *Cell Calcium*, 56, 493-503.

Sarraj, M.A., McClive, P.J., Szczepny, A., Daggag, H., Loveland, K.L., & Sinclair, A.H. (2007). Expression of *Wsb2* in the developing and adult mouse testis. *Reproduction*, 133, 753-761.

Schmidt, P.S., Zhu, C-T., Das, J., Batavia, M., Yang, L., & Eanes, W.F. (2008). An amino acid polymorphism in the couch potato gene forms the basis for climatic adaptation in Drosophila melanogaster. *Proceedings of the National Academy of Sciences of the United States of America*, 105, 16207-16211.

Schoenauer, R., Lange, S., Hirschy, A., Ehler, E., Perriard, J.-C., & Agarkova, I. (2008). Myomesin 3, a novel structural component of the M-band in striated muscle. *Journal of Molecular Biology*, 376, 338-351.

Shafqat, N., Kavanagh, K.L., Sass, J.O., Christensen, E., Fukao, T., Lee, W.H., Oppermann, U., & Yue, W.W. (2013). A structural mapping of mutations causing succinyl-CoA: 3-ketoacid CoA transferase (SCOT) deficiency. *Journal of Inherited Metabolic Disease*, 36, 983–987.

Sham, H.P., Yu, E.Y.S., Gulen, M.F., Bhinder, G., Stahl, M., Chan, J.M., …Vallance, B.A. (2013). SIGIRR, a negative regulator of TLR/IL-1R signalling promotes microbiota dependent resistance to colonization by enteric bacterial pathogens. *PLoS Pathogens*, 9(8), e1003539.

Sherag, A., Dina, C., Hinney, A., Vatin, V., Scherag, S., Vogel, C.I.G., … Meyre, D. (2010). Two new loci for body-weight regulation identified in a joint analysis of genome-wide association studies for early-onset extreme obesity in French and German study groups. *PLoS Genetics*, 6(4), e1000916.

Shibuya, K., Obayashi, I., Asakawa, S., Minoshima, S., Kudoh, J., & Shimizu, N. (2004). A cluster of 21 keratin-associated protein genes within introns of another gene on human chromosome 21q22.3. *Genomics*, 83, 679-693.

Sironen, A., Thomsen, B., Andersson, M., Ahola, V., & Vilkki, J. (2006). An intronic insertion in *KPL2* results in aberrant splicing and causes the immotile short-tail sperm defect in the pig. *Proceedings of the National Academy of Sciences of the United States of America*, 103, 5006-5011.,

Strazielle, C., Lefevre, A., Jacquelin, C., & Lalonde, R. (2012). Abnormal grooming activity in *Dab1^scm^* (*scambler*) mutant mice. *Behavioural Brain Research*, 233, 24-28.

Sugimoto, M., Gotoh, Y., Kawahara, T., & Sugimoto, Y. (2015). Molecular effects of polymorphism in the 3’UTR of *Unc-5 homolog C* associated with conception rate in Holsteins. *PLoS ONE*, 10(7), e0131283.

Sugimoto, M., Sasaki, S., Gotoh, Y., Nakamura, Y., Aoyagi, Y., Kawahara, T., & Sugimoto, Y. (2013). Genetic variants related to gap junctions and hormone secretion influence conception rates in cows. *Proceedings of the National Academy of Sciences of the United States of America*, 110, 19495-19500.

Sun, C., Southard, C., Witonsky, D.B., Kittler, R., & Di Rienzo. A. (2010). Allele-specific down-regulation of RPTOR expression induced by retinoids contributes to climate adaptations. *PLoS Genetics*, 6(10), e1001178.

Sun, N., Chen, Y., Peng, H., Luo, Y., & Zhang, G. (2016). A novel Ala275Val mutation in factor X gene influences its structural compatibility and impairs intracellular trafficking and coagulant activity. *Thrombosis Research*, 138, 108-113.

Takasuga, A., Sato, K., Nakamura, R., Saito, Y., Sasaki, S., Tsuji, T.,… Kojima, T. (2015). Non-synonymous FGD3 variant as positional candidate for disproportional tall stature accounting for a carcass weight QTL (*CW-3*) and skeletal dysplasia in Japanese black cattle. *PLoS Genetics*, 11(8), e1005433.

Talaei, F., Schmidt, M., & Henning, R.H. (2011). Induction of VMAT-1 and TPH-1 expression induces vesicular accumulation of serotonin and protects cells and tissue from cooling/rewarming injury. *PLoS ONE*, 7(1): e30400.

Tetsuka, M., Nishimoto, H., Miyamoto, A., Okuda, K., & Hamano, S. (2010). Gene expression of 11β-HSD ad glucocorticoid receptor in the bovine (*Bos taurus*) follicle during follicular maturation and atresia: the role of follicular stimulating hormone. *Journal of Reproduction and Development*, 56, 616-622.

Tominaga, K., Kondo, C., Kagata, T., Hishida, T., Nishizuka, M., & Imagawa, M. (2004). The novel gene fad158, having a transmembrane domain and leucine-rich repeat, stimulates adipocyte differentiation. *The Journal of Biological Chemistry*, 279, 34840-34848.

Tong, B., Sasaki, S., Muramatsu, Y., Ohta, T., Kose, H., Yamashiro, H., Fujita, T., & Yamada, T. (2014). Association of a single-nucleotide polymorphism in *myosin-binding protein C, slow-type (MYBPC1)* gene with marbling in Japanese Black beef cattle. *Animal Genetics*, 45, 609-613.

Tong, B., Xing, Y.P., Muramatsu, Y., Ohta, T., Kose, H., Zhou, H.M., & Yamada, T. (2015). Association of expression levels in skeletal muscle and a SNP in the *MYBPC1* gene with growth-related trait in Japanese Black beef cattle. *Journal of Genetics*, 94, 135-137.

Townson, S.M., Dobrzyck, K.M., Lee, A.V., Air, M., Deng, W., Kang, K., & Oesterreich, S. (2003). SAFB2, a new scaffold attachment factor homolog and estrogen receptor corepressor. *The Journal of Biological Chemistry*, 278, 20059–20068.

Urano, T., Shiraki, M., Sasaki, N., Ouchi, Y., & Inoue, S. (2014). Large-scale analysis reveals a functional single-nucleotide polymorphism in the 5’-flanking region of *PRDM16* gene associated with lean body mass. *Aging Cell*, 13, 739-743.

Vallet, M., Soares, D.C., Wani, S., Sophocleous, A., Warner, J., Salter, D.M., Ralston, S.H., & Albagha, O.M.E. (2015). Targeted sequencing of the Paget’s disease associated 14q32 locus identifies several missense coding variants in *RIN3* that predispose to Paget’s disease of bone. *Human Molecular Genetics*, 24, 3286-3295.

Van der Valk, R.J.P. Kreiner-Møller, E., Kooijman, M.N., Guxens, M., Stergiakouli, E., & Sääf, A. (2015). A novel common variant in DCST2 is associated with length in early life and height in adulthood. *Human Molecular Genetics*, 24, 1155-1168.

Wang, J, Wang, C., Tian, R., Huang, Y.-Z., Lai, X.-S., Lan, X.-Y., Wang, J.-Q., & Chen, H. (2012). Sequence variants in the bovine *PRDM16* gene associated with body weight in Chinese cattle breeds. *Genetics and Molecular Research*, 11, 746-755.

Wei, W., He, H.-B., Zhang, W.-Y., Zhang, H.-X., Bai, J.-B., Liu, H.-Z., Cao, J.-H., Chang, K.C., Li, X.-Y., & Zhao, S.-H. (2013). miR-29 targets Akt3 to reduce proliferation and facilitate differentiation of myoblasts in skeletal muscle development. *Cell Death and Disease*, 4, e668.

Winter, H., Hofmann, I., Langbein, L., Rogers, M.A., & Schweizer, J. (1997). A splice site mutation in the gene of the human type I hair keratin hHa1 results in the expression of a tailless keratin isoform. *The Journal of Biological Chemistry*, 51, 32345–32352.

Won, J., de Evsikova, C.M., Smith, R.S., Hicks, W.L., Edwards, M.M., Longo-Guess, C.,… Nishina, P.M. (2011). NPHP4 is necessary for normal photoreceptor ribbon synapse maintenance and outer segment formation, and for sperm development. *Human Molecular Genetics*, 20, 482-496.

Wu, D.-D., Irwin, D.M., & Zhang, Y.-P. (2008). Molecular evolution of the keratin associated protein gene family in mammals, role in the evolution of mammalian hair. *BMC Evolutionary Biology*, 8, 241.

Xu, H., Furman, M., Mineur, Y.S., Chen, H., King, S.L., Zenisek, D., ... Crair, M.C. (2011). An instructive role for patterned spontaneous retinal activity in mouse visual map development. *Neuron*, 70, 1115–1127.

Xu, X.R., Wang, J.J., Yang, Q.Y., Jiao, J., He, L.H., Yu, S.F., … Jin, X. N. (2017). The effect of PCDH15 gene variations on the risk of noise-induced hearing loss in a Chinese population. *Biomedical and Environmental Sciences*, 30, 143-146.

Xu, W., Chen, Q., Liu, C., Chen, J., Xiong, F., & Wu, B. (2017). A novel, complex RUNX2 gene mutation causes cleidocranial dysplasia. *BMC Medical Genetics*,18,13.

Yamamoto, Y., Yamada, S., Kodera, T., Hara, A., Motoyoshi, K., Tanaka, Y., …Kojima, I. (2008). Reversal of streptozotocin-induced hyperglycemia by continuous supply of betacellulin in mice, *Growth Factors*, 26, 173-179.

Zahoor, M. A., Yamane, D., Mohamed, Y. M., Suda, Y., Kobayashi, K., Kato, K., Tohya, Y., & Akashi, H. (2010). Bovine viral diarrhea virus non-structural protein 5A interacts with NIK- and IKKβ-binding protein. *Journal of General Virology*, 91, 1939–1948.

Zhang, G., Feenstra, B., Bacelis, J., Liu, X., Muglia, L.M., Juodakis, J., …Muglia, L.J. (2017). Genetic associations with gestational duration and spontaneous preterm birth. *The New England Journal of Medicine*, 377, 1156-1167.

Zhang, J.-X., Wang, C., Yang, C.-Y., Wang, J.-Y., Chen, L., Bao, X.-M., … Liu, J. (2010). The role of arabidopsis *AtFes1A* in cytosolic Hsp70 stability and abiotic stress tolerance. *The Plant Journal*, 62, 539-548.

Zhang, L.-Z., Ren, G., Hua, L.-S., Lan, X.-Y., lei, C.-Z., & Chen, H. (2014). Polymorphism in the 5’-UTR of the insulin-like growth factor I gene associated with production traits in Chinese cattle. *Genetics and Molecular Research*, 13, 6899-6905.

Zhang, S., Pondarre, C., Pennarun, G., Labussiere‑Wallet, H., Vera, G., France, B., … de Villartay, J.-P. (2016). A nonsense mutation in the DNA repair factor Hebo causes mild bone marrow failure and microcephaly. *Journal of Experimental Medicine*, 213, 1011-1028.

Zhao, F., Liu, C., Hao, Y.-M-, Qu, B., Cui, Y.-J., Zhang, N., Gao, X.-J., & Li, Q.-Z. (2015). Up-regulation of integrin α6β4 expression by mitogens involved in dairy cow mammary development. *In Vitro Cellular & Developmental Biology – Animal*, 51, 287-299.

Zhao, F., Zheng, Y.-C., & McFadden, T.B. (2005). Cloning and expression of bovine sodium/glucose cotransporters. *Journal of Dairy Science*, 88,182–194.

Zhao, M., Qiao, M., Harris, S.E., Oyajobi, B.O., Mundy, G.R., & Chen, D. (2004). Smurf1 inhibits osteoblast differentiation and bone formation *in vitro* and *in vivo*. *Journal of Biological Chemistry*, 279, 12854-12859.

Zhou, G.-B., Liu, G.-S., Meng, Q.-G., Liu, Y., Hou, Y.-P., Wang, X.-X., Li, N., & Zhu, S.-E. (2009). Tetraspanin CD9 in bovine oocytes and its role in fertilization. *Journal of Reproduction and Development*, 55, 305-308.

Zhou, G.-B., Zeng, Y., Meng, Q.-G., Liu, Y., Dai, Y.-P., Zhu, S.-E., Bunch, T.D., & Hou, Y.-P. (2013). Decreased expression of CD9 in bovine oocytes after cryopreservation and the relationship to fertilization capacity. *Molecular Reproduction & Development*, 80, 451-459.

**Supplemental Table S5**. Excel file with results for enrichment analyses detailing Gene Ontology (GO) term ID, term type, adjusted p-value, term group, term depth, term name, and the number of genes in a term (T) that overlaps with recognized genes in the input query (Q), denoted Q&T list.

**Figures**

**
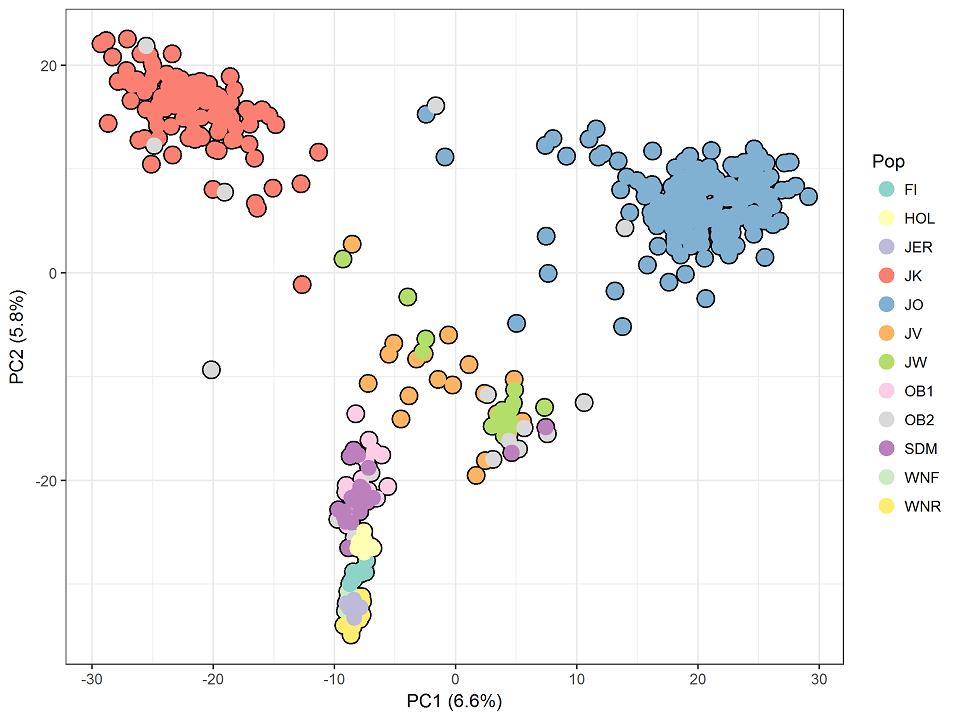
**

**Supplemental Figure S1.** Principal component analyses with 472 individuals showing the first and second PC axes, including all sampled individuals for the two larger of the four contemporary Jutland lineages, Kortegaard (n = 131) and Oregaard (n = 186). Cattle breeds/lineages (denoted in figure legend as Pop) are FI: Faroe Island cattle, HOL: Holstein, JER: Jersey, JK: Jutland cattle Kortegaard-lineage, JO: Jutland cattle Oregaard-lineage, JV: Jutland cattle Vesterbølle-lineage, JW: Jutland cattle Westergaard-lineage, OB1: Old bulls pre-1980 (cryopreserved semen samples from SDM-1965 cattle), OB2: Old bulls post-1980 (cryopreserved from n=14 Jutland and n=4 SDM-1965 cattle), SDM: SDM-1965 cattle, WNF: Western Norwegian Fjord cattle, WNR: Western Norwegian Red-polled cattle.

a)


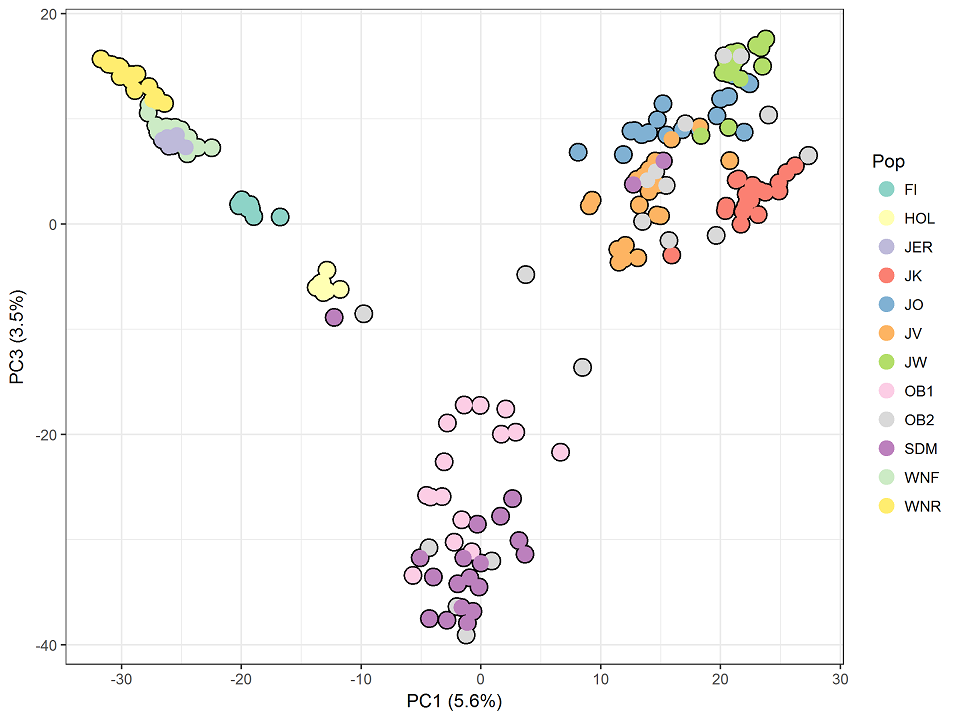


b)


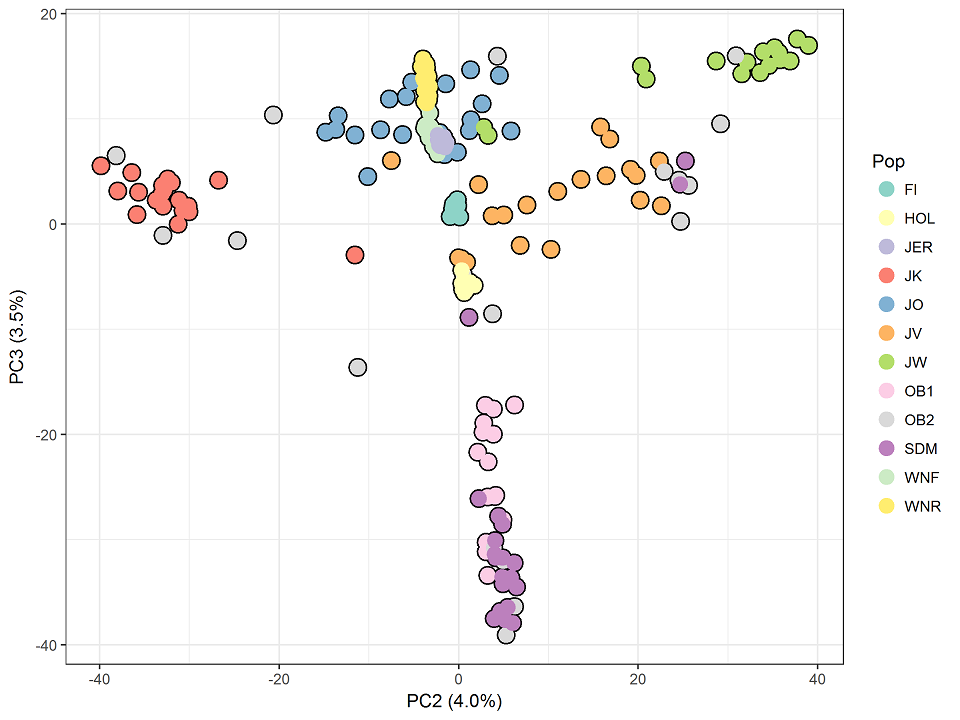


c)


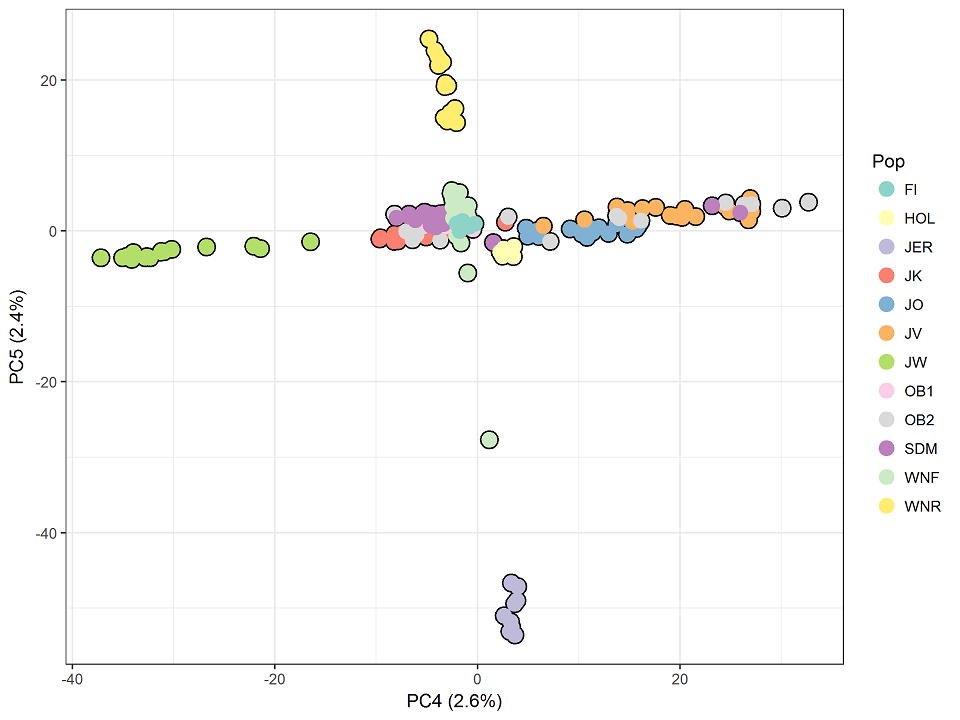


**Supplemental Figure S2.** Principal Component Analyses (PCA) with 195 individuals showing (a) the first and third PC axes, (b) the third and fourth PC axes, and (c) the fourth and fifth PC axes. Cattle breeds/lineages (denoted in figure legend as Pop) are FI: Faroe Island cattle, HOL: Holstein, JER: Jersey, JK: Jutland cattle Kortegaard-lineage, JO: Jutland cattle Oregaard-lineage, JV: Jutland cattle Vesterbølle-lineage, JW: Jutland cattle Westergaard-lineage, OB1: Old bulls pre-1980 (cryopreserved semen samples from SDM-1965 cattle), OB2: Old bulls post-1980 (cryopreserved from n=14 Jutland and n=4 SDM-1965 cattle), SDM: SDM-1965 cattle, WNF: Western Norwegian Fjord cattle, WNR: Western Norwegian Red-polled cattle.

(a) Kortegaard (b) Oregaard


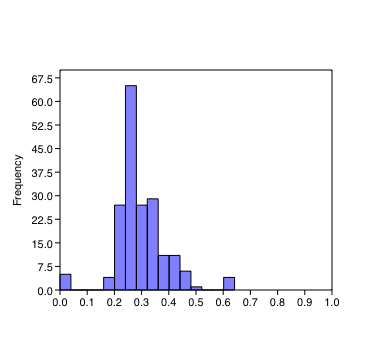

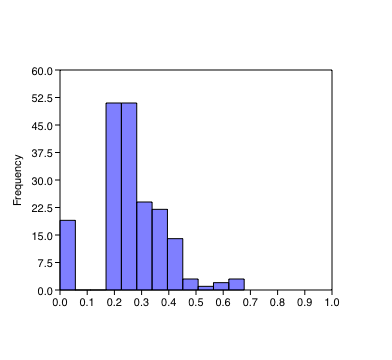


(c) Vesterbølle (d) Westergaard


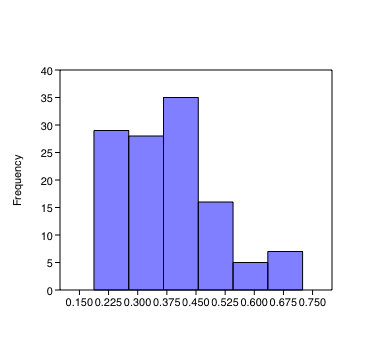

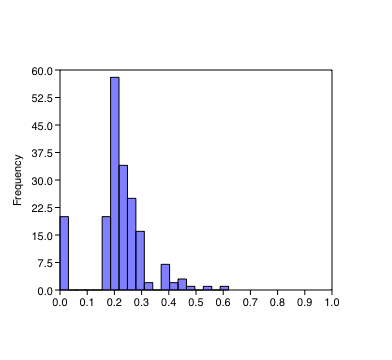


(e) Old bulls pre-1980 (f) Old bulls post-1980


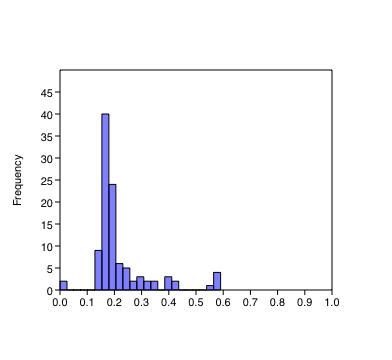

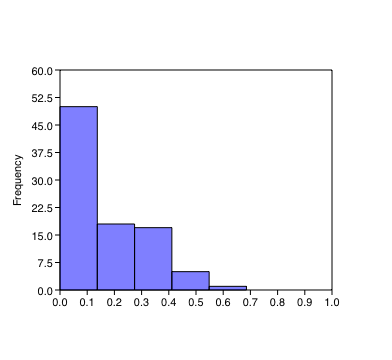


(g) SDM-1965 (h) Western Norwegian Fjord cattle


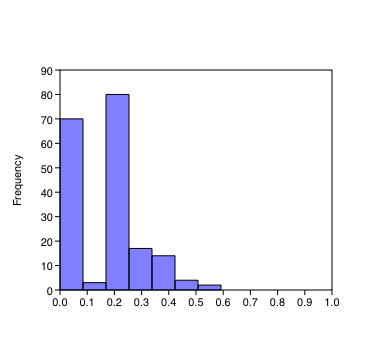

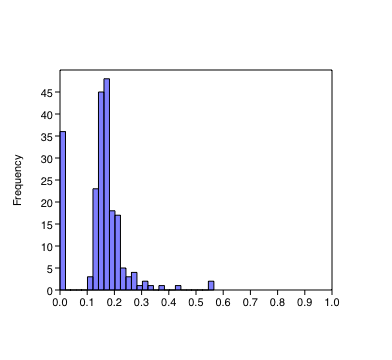


(i) Western Norwegian Red-polled cattle (j) Faroe Island cattle
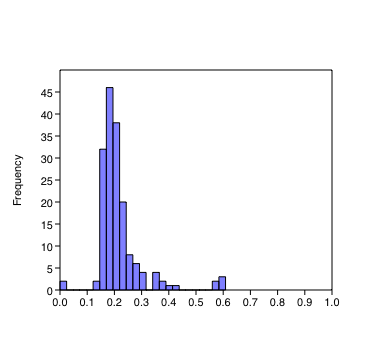

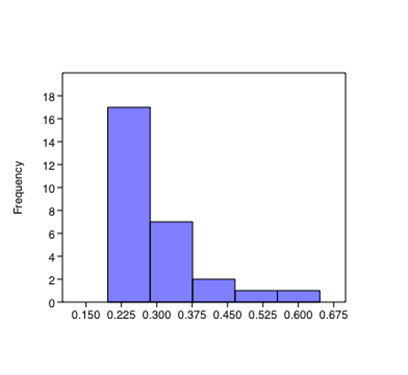


(k) Holstein (l) Jersey


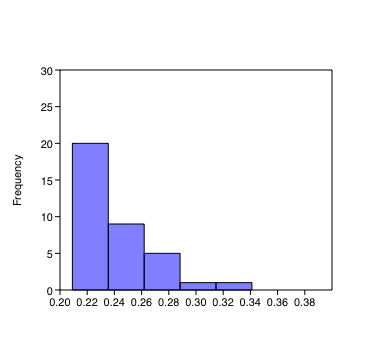

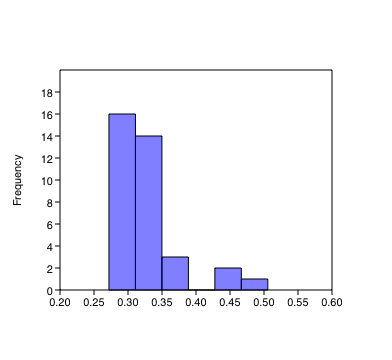


**Supplemental Figure S3**. Identity-By-Descent (IBD) frequency plots for cattle breeds and lineages. The horizontal axis shows the IBD values (theoretical range 0-1).
